# Heterogeneity analysis of the proteomes in clinically nonfunctional pituitary adenomas

**DOI:** 10.1186/s12920-014-0069-6

**Published:** 2014-12-24

**Authors:** Xianquan Zhan, Xiaowei Wang, Ying Long, Dominic M Desiderio

**Affiliations:** Key Laboratory of Cancer Proteomics of Chinese Ministry of Health, Xiangya Hospital, Central South University, 87 Xiangya Road, Changsha, Hunan 410008 P. R. China; Hunan Engineering Laboratory for Structural Biology and Drug Design, Xiangya Hospital, Central South University, 87 Xiangya Road, Changsha, Hunan 410008 P. R. China; State Local Joint Engineering Laboratory for Anticancer Drugs, Xiangya Hospital, Central South University, 87 Xiangya Road, Changsha, Hunan 410008 P. R. China; The State Key Laboratory of Medical Genetics, Central South University, 88 Xiangya Road, Changsha, Hunan 410008 P. R. China; The Charles B. Stout Neuroscience Mass Spectrometry Laboratory, Department of Neurology, College of Medicine, University of Tennessee Health Science Center, 847 Monroe Avenue, Memphis, TN 38163 USA

**Keywords:** Nonfunctional pituitary adenoma, Proteome, Heterogeneity, Two-dimensional gel electrophoresis, Mass spectrometry, Differentially expressed protein, Pathway network

## Abstract

**Background:**

Clinically nonfunctional pituitary adenomas (NFPAs) without any clinical elevation of hormone and with a difficulty in its early-stage diagnosis are highly heterogeneous with different hormone expressions in NFPA tissues, including luteinizing hormone (LH)-positive, follicle-stimulating hormone (FSH)-positive, LH/FSH-positive, and negative (NF). Elucidation of molecular mechanisms and discovery of biomarkers common and specific to those different subtypes of NFPAs will benefit NFPA patients in early-stage diagnosis and individualized treatment.

**Methods:**

Two-dimensional gel electrophoresis (2DGE) and PDQuest image analyses were used to compare proteomes of different NFPA subtypes (NF-, LH-, FSH-, and LH/FSH-positive) relative to control pituitaries (Con). Differentially expressed proteins (DEPs) were characterized with mass spectrometry (MS). Each set of DEPs in four NFPA subtypes was evaluated with overlap analysis and signaling pathway network analysis with comparison to determine any DEP and pathway network that are common and specific to each NFPA subtype.

**Results:**

A total of 93 differential protein-spots were determined with comparison of each NFPA type (NF-, LH-, FSH-, and LH/FSH-positive) versus control pituitaries. A total of 76 protein-spots were MS-identified (59 DEPs in NF vs. Con; 65 DEPs in LH vs. Con; 63 DEPs in FSH vs. Con; and 55 DEPs in LH/FSH vs. Con). A set of DEPs and pathway network data were common and specific to each NFPA subtype. Four important common pathway systems included MAPK-signaling abnormality, oxidative stress, mitochondrial dysfunction, and cell-cycle dysregulation. However, these pathway systems were, in fact, different among four NFPA subtypes with different protein-expression levels of most of nodes, different protein profiles, and different pathway network profiles.

**Conclusions:**

These result data demonstrate that common and specific DEPs and pathway networks exist in four NFPA subtypes, and clarify proteome heterogeneity of four NFPA subtypes. Those findings will help to elucidate molecular mechanisms of NFPAs, and discover protein biomarkers to effectively manage NFPA patients towards personalized medicine.

**Electronic supplementary material:**

The online version of this article (doi:10.1186/s12920-014-0069-6) contains supplementary material, which is available to authorized users.

## Background

Clinically nonfunctional pituitary adenomas (NFPAs) are a very challenging clinical problem in pituitary tumor patients relative to functional pituitary adenomas (FPAs) because an NFPA does not have any elevation of the corresponding hormone [1,2]. Thus, an NFPA commonly cannot be diagnosed until presentation of visual injuries and compression symptoms of neighboring tissues, when the tumor has progressed to the middle/late stage. An opportunity is lost for early-stage treatment, and central endocrine regulatory roles of the pituitary, are both lost. The use of proteomics to elucidate molecular mechanisms and discover tumor-related NFPA biomarkers is our long-term goal. Extensive proteomics studies of pituitary adenomas have been carried out in our, and other, research groups [3-10], including protein expression profiles [11-14], differentially expressed proteins (DEPs) [15,16], protein post-translational modifications (PTMs) that include tyrosine nitration [17-19] and phosphorylation [20,21], hormone isoforms [22], protein molecular pathway networks [16,23] from comparative proteomics and systems biology analyses between NFPA versus control tissues [15,23] and between invasive versus noninvasive NFPAs [16], and serum protein biomarkers in pituitary adenomas [6-8]. Moreover, a protein antibody array (n = 1,005 proteins) based on pituitary adenoma proteomics data was used to analyze human pituitary adenomas and identify a DEP profile [24]. A clinical proteomic method that was used to accurately stratify pituitary adenomas was based on multiplex immunoassays of peptide hormones extracted from formalin-fixed and paraffin-embedded tissue [9,10]. Laser capture microdissection (LCM) coupled with proteomics was used to accurately identify the proteomic variation of an adenoma relative to control pituitary [25-27]. Proteomics data-based systems pathway network analysis revealed four important signal pathway network variations in NFPA pathophysiological processes, including mitochondrial dysfunction, oxidative stress, cell-cycle dysregulation, and MAPK-signaling system abnormality [23]. However, those proteomic studies did not consider NFPA heterogeneity. In fact, NFPA is highly heterogeneous – it has different types of cell origins and hormones expressed in tumor tissues, including silent somatotroph (growth hormone GH-positive; 3%), silent corticotroph (adrenocorticotropic hormone ACTH-positive; 8%), oncocytoma (no hormone expression; 6%), null cell (no hormone expression; 17%), and gonadotroph (intact follicle-stimulating hormone/luteinizing hormone FSH/LH or subunits; 40-79%) that was subclassified as LH-positive, FSH-positive, and LH/FSH-positive NFPAs [15]. Studies demonstrate that the silent hormone expression in NFPA tissue is associated with tumor behaviors such as invasion [28,29]. Therefore, it is essential to investigate proteomic variations in different NFPA subtypes for an in-depth and accurate understanding of common and specific NPFA mechanisms and discovery of reliable biomarkers toward individualized medical practice. Thus, we emphasize the scientific importance to investigate the heterogeneity of NPFA proteomes. A comparative analysis of proteomes in different NFPA subtypes (NF-, LH-, FSH-, and LH/FSH-positive) relative to normal control pituitaries described here revealed variations in protein expressions and pathway networks among four NFPA subtypes, and revealed the common and specific molecular mechanisms and protein biomarkers for different NFPA subtypes.

**Scheme 1:**
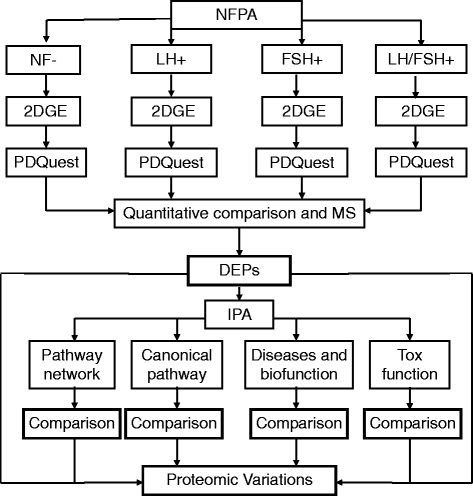
**Overall experimental flow-chart used to analyze the proteomic variations among different NFPA subtypes (NF-, LH-, FSH-, and LH/FSH-).** NFPA, nonfunctional pituitary adenoma; 2DGE, two-dimensional gel electrophoresis; DEP, differentially expressed proteins; MS, mass spectrometry; and IPA, Ingenuity Pathway Analysis.

Two-dimensional gel electrophoresis (2DGE)-based comparative proteomics [30,31] is a classical and effective approach to quantify each DEP in different NFPA subtypes (NF-, LH-, FSH-, and LH/FSH-positive) versus normal control pituitaries. The linear dynamic separation range, spatial and quantitative reproducibility, and experimental conditions of 2DGE-based proteomics were optimized in our previous studies [32,33]. Isoelectric focusing (IEF) has a high reproducibility due to use of highly reproducible commercial immobilized pH gradient (IPG) strip. The vertical multi-gel sodium dodecyl sulfate–polyacrylamide gel electrophoresis (SDS-PAGE) system, which has a higher reproducibility and wider linear dynamic range, was used to array the proteome of each NFPA subtype and control pituitary. PDQuest 2D image software was used to quantify DEPs of each NFPA subtype relative to controls. Matrix-assisted laser desorption/ionization time-of-flight (MALDI-TOF)-based peptide mass fingerprint (PMF), liquid chromatography (LC)-electropray ionization (ESI)-based tandem mass spectrometry (MS/MS), and human Swiss-Prot protein databases were used to identify each DEP. Systems biology-based pathway network analysis was used to reveal variations of protein molecular networks among different NFPA subtypes. Thus, this study is the first to reveal variations in proteomes and molecular networks among four NFPA subtypes (NF-, LH-, FSH-, LH/FSH-positive NFPAs), and the heterogeneity of NFPA proteomes. Scheme 1 shows the overall experimental flow-chart that was used to identify variations of proteomes and molecular networks among four NFPA subtypes.

## Results

### 2DGE pattern and DEP profile of each NFPA subtype relative to control pituitaries

**Figure 1:**
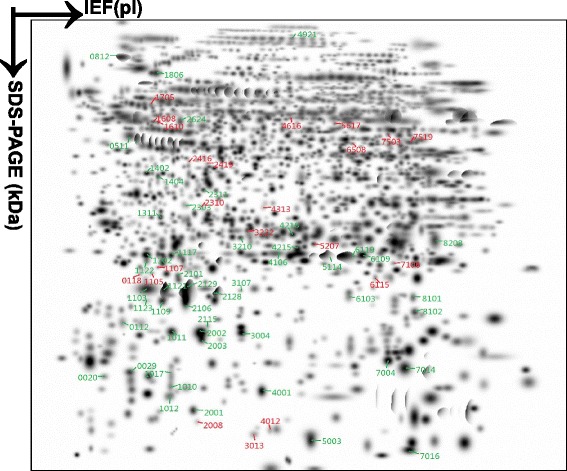
**Two-dimensional gel electrophoresis reference map from a human pituitary control proteome labeled with 72 differential spots among different NFPA subtypes (NF-, LH-, FSH-, and LH/FSH-).** The isoelectric focusing (IEF) was carried out with an 18-cm IPG strip (pH 3–10 NL), and a vertical SDS-PAGE with a 12% polyacrylamide gel.

2DGE-based comparative proteomics was used to analyze proteome variations from controls (n = 8; 3 to 5 gel images per sample) versus each NFPA type, including NF- (n = 3; 3 gel images per sample), LH- (n = 3; 3 gel images per sample), FSH- (n = 3; 3 gel images per sample), and LH/FSH- (n = 3; 3 gel images per sample) adenoma tissues. Each sample was 2DGE-analyzed three to five times to provide a triplicate, high-quality 2DGE gel image for each sample. Figure 1 shows the digitized master 2D gel image. *Ca.* 1,000 protein-spots were detected in each gel image with PDQuest 2D gel-image analysis with high-resolution, high-spatial reproducibility in the IEF and SDS-PAGE directions, and high reproducibility for triplicate 2D gel images of each sample. 2DGE protein-spots mainly distributed within pH 4–9 and relative mass (M_r_) 15–150 kDa. For each sample, the average between-gel matched percentage ranged from 85% to 99% for control pituitaries and 81% to 90% for NFPAs; the positional deviation of matched spots among triplicate 2D gel images was 2.13 ± 0.79 mm in the IEF direction and 1.82 ± 0.68 mm in the SDS-PAGE direction; and the correlation coefficient (r) of normalized spot-volumes for between-gel matched spots was > 0.76 with a range of 0.76-0.92. The high-spatial reproducibility and quantitative reproducibility resulted from the same experimental condition maintained for each sample analysis, including sample preparation, IEF, SDS-PAGE, visualization, gel digitalization, and image analysis. That reproducibility provided an accurate comparison between each NFPA subtype and controls.

**Figure 2:**
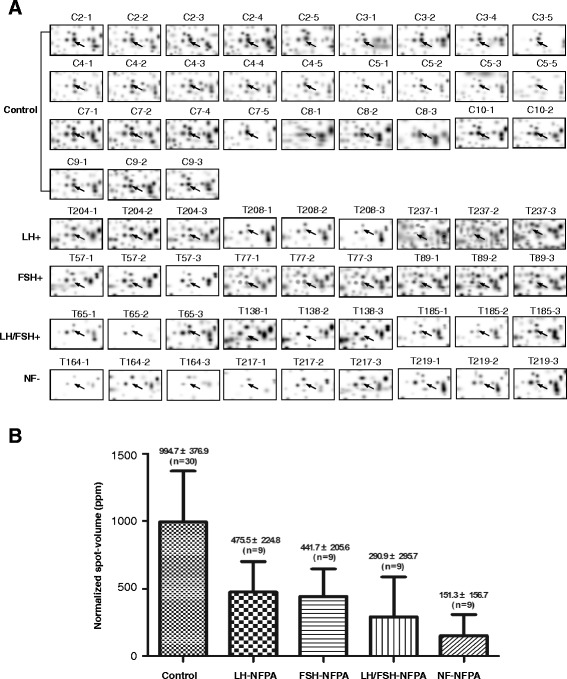
**A representative differential spot among different NFPA subtypes versus control pituitaries (Spot-1311).** Control means control pituitaries; LH means LH-positive NFPA; FSH means FSH-positive NFPA; LH/FSH means LH/FSH-positive NFPA; and NF means NFPA with negative hormone expression. A statistically significant difference existed between each type of NFPAs versus control pituitaries (p < 0.001). **A.** The 2DGE images (Spot-1311) among four NFPA subtypes. **B.** The normalized spot-volume (Spot-1311) among four NFPA subtypes.

**Figure 3:**
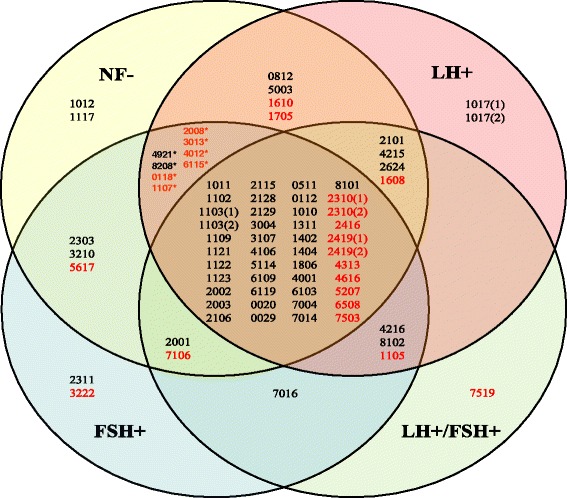
**Overlap analysis of differentially expressed proteins among four NFPA subtypes (NF-, LH-, FSH-, and LH/FSH-).** Each number in this figure is corresponded to the SSP number in the Table 1. Those duplicated SSP Numbers in the Table 1 are re-labeled in this figure as following: 1017(1) = Chain 1, somatotropin; 1017(2) = Splice isoform 2 of somatotropin precursor; 1103(1) = Somatotropin precursor; 1103(2) = Growth hormone variant precursor; 2310(1) = F-actin capping protein beta subunit; 2310(2) = Splice isoform 2 of F-actin capping protein beta subunit; 2419(1) = Guanine nucleoide-binding protein G(O), alpha subunit 1; and 2419(2) = Guanine nucleotide-binding protein G(O), alpha subunit 2. The red color number refers to an upregulated protein. The black color number refers to a downregulated protein. The label (*) means those DEP-spots are common to only two groups of LH- and FSH-NFPA, not to NF-NFPA.

A total of 93 differential protein-spots was determined with comparison of each NFPA subtype (NF-, LH-, FSH-, and LH/FSH-) versus control pituitaries with a cut-off value of >3-fold and a statistically significant difference (p < 0.001). Figure 2 shows a representative DEP-spot (Spot-1311) among different NFPA subtypes (n = 9 gel images for each type of NFPAs) versus controls (n = 30 gel images) (Figure 2A) and a quantitative comparison of normalized spot-volumes between each NFPA subtype versus controls (p < 0.001) (Figure 2B). Each differential protein-spot was labeled in the master gel image (Figure 1). Each differential protein-spot was excised, and proteins were subjected to in-gel digestion with trypsin, followed by MS analysis, including MALDI-TOF PMF and LC-ESI-MS/MS. A total of 72 differential protein-spots that contained DEPs were MS-identified from those 93 differential protein-spots. Four spots (SSP No. 1017, 1103, 2310, and 2419 in Table 1 and Figure 3) of 72 MS-identified spots contained two proteins; therefore, 72 spots represent 76 protein-spots. Among those 76 protein-spots, 59 DEPs (44 downregulated and 15 upregulated) were identified in NF-NFPA relative to controls, 65 DEPs (45 downregulated and 20 upregulated) in LH-NFPA, 63 DEPs (43 downregulated and 20 upregulated) in FSH-NFPA, and 55 DEPs (41 downregulated and 14 upregulated) in LH/FSH-NFPA. Those DEPs are summarized in Table 1, which contains the SSP number that corresponds to the number labeled in Figure 1, protein name, Swiss-Prot accession number, and fold-value in each NFPA subtype versus controls.

**Table 1:** **Differentially expressed proteins in different subtypes of clinically nonfunctional pituitary adenomas versus controls**

**SSP no.**	**Protein name**	**Swiss-Prot no.**	**NF-NFPA**	**LH-NFPA**	**FSH-NFPA**	**LH/FSH-NFPA**
1011	Chain 1: somatotropin	P01241	180.6(−)	44.1(−)	82.6(−)	90.5(−)
1017	Chain 1: somatotropin	P01241		13.3(−)		
1017	Splice isoform 2 of somatotropin precursor	P01241 isoform		13.3(−)		
1102	Chain 1: somatotropin	P01241	20.2(−)	6.5(−)	9.1(−)	9.3(−)
1103	Somatotropin precursor	P01241	14.1(−)	2.9(−)	3.3(−)	5.8(−)
1103	Growth hormone variant precursor	P01242	14.1(−)	2.9(−)	3.3(−)	5.8(−)
1109	Chain 1: somatotropin	P01241 isoform	(−−)	128.3(−)	69.4(−)	(−−)
1121	Chain 1: somatotropin	P01241	6.2(−)	2.8(−)	2.7(−)	3.3(−)
1122	Chain 1: somatotropin	P01241	48.1(−)	(−−)	7.5(−)	8.1(−)
1123	Chain 1: somatotropin	P01241	17.3(−)	4.4(−)	6.4(−)	(−−)
2002	Splice isoform 2 of somatotropin precursor	P01241 isoform	9.7(−)	3.9(−)	3.2(−)	5.4(−)
2003	Chain 1: somatotropin	P01241	(−−)	88.8(−)	25.0(−)	(−−)
2101	Chain 1: somatotropin	P01241	27.8(−)	(−−)		17.4(−)
2106	Chain 1: somatotropin	P01241	(−−)	9.4(−)	2.2(−)	51.2(−)
2115	Chain 1: somatotropin	P01241	(−−)	32.1(−)	(−−)	(−−)
2128	Chain 1: somatotropin	P01241	(−−)	82.1(−)	67.4(−)	478.6(−)
2129	Chain 1: somatotropin	P01241	13.4(−)	6.7(−)	4.3(−)	5.5(−)
3004	Chain 1: somatotropin	P01241	42.0(−)	37.0(−)	21.2(−)	13.6(−)
3107	Chain 1: somatotropin	P01241	30.5(−)	(−−)	19.1(−)	34.9(−)
4106	Chain 1: prolactin	P01236	8.3(−)	12.6(−)	46.2(−)	99.9(−)
4215	Prolactin precursor	P01236	4.9(−)	4.1(−)		3.8(−)
4216	Chain 1: prolactin	P01236		26.2(−)	14.6(−)	12.3(−)
5114	Chain 1: prolactin	P01236	(−−)	20.1(−)	17.6(−)	19.0(−)
6109	Chain 1: prolactin	P01236	(−−)	36.7(−)	(−−)	19.7(−)
6119	Chain 1: prolactin	P01236	(−−)	33.6(−)	11.3(−)	32.6(−)
0020	Splice isoform IL15-S21AA of interleukin-15 precursor	P40933-2 isoform	(−−)	(−−)	1.6(−)	2.7(−)
0029	Chain 1: Factor X light chain	P00742	(−−)	(−−)	29.3(−)	38.9(−)
0511	Cytokeratin 16	P08779	28.8(−)	2.1(−)	15.7(−)	18.5(−)
0112	Alpha crystallin C chain	Q9UJY1	(−−)	2.3(−)	(−−)	(−−)
0812	Chain 1: Endoplasmin (Tumor rejection antigen 1)	P14625	11.0(−)	4.4(−)		
1010	14-3-3 protein tau	P27348	(−−)	(−−)	6.4(−)	15.5(−)
1012	Splice isoform 3 of alpha-s1 casein precursor	P47710-3 isoform	17.8(−)			
1117	Chain 1: apolipoprotein A-I	P02647	2.1(−)			
1311	Secretagogin	O76038	6.6(−)	2.1(−)	2.3(−)	3.4(−)
1402	Mu-crystallin homolog	Q14894	16.3(−)	4.6(−)	2.4(−)	4.6(−)
1404	Chain 1: MIMECAN	P20774	38.0(−)	6.1(−)	9.1(−)	16.2(−)
1806	Tissue transglutaminase	P21980	(−−)	17.1(−)	(−−)	(−−)
2001	CD59 glycoprotein	P13987	9.5(−)		2.4(−)	4.1(−)
2303	Serine/threonine protein phosphatase 2A, 55 kDa regulatory subunit B, alpha isoform	Q00007	8.2(−)		2.6(−)	
2311	L-Myc-1 protooncogene protein	P12524			7.4(−)	
2624	Chain 1: dipeptidyl-peptidase II	Q9UHL4	9.3(−)	2.5(−)		5.3(−)
3210	Heat shock protein 27	P04792	2.4(−)		4.9(−)	
4001	ATP binding protein associated with cell differentiation	O14530	11.4(−)	2.3(−)	2.7(−)	4.4(−)
4921	Chain 1: collagen alpha 2 (VI) chain	P12110		5.3(−)	14.7(−)	
5003	Hemoglobin beta-2 chain	P18988	3.1(−)	2.1(−)		
6103	N6-adenosine-methyltransferase 70 kDa subunit	Q86U44	6.2(−)	6.5(−)	(−−)	8.3(−)
7004	Hemoglobin beta unit variant	gi1066765	(−−)	176.7(−)	(−−)	(−−)
7014	Hemoglobin beta-2 chain	P18988	(−−)	29.4(−)	28.9(−)	99.4(−)
7016	Hemoglobin alpha-2 chain	P01968			20.2(−)	13.3(−)
8101	Chain 1: insulin-like growth factor binding	P24592	(−−)	2.9(−)	20.9(−)	5.0(−)
8102	Chain 1: phospholipid hydroperoxide glutathione peroxidase	P36969		3.9(−)	2.1(−)	14.4(−)
8208	Ig kappa chain V-III region SIE	P01620		4.1(−)	32.5(−)	
0118	Lactoylglutathione lyase	Q04760		9.1(+)	10.2(+)	
1105	Lactoylglutathione lyase	Q04760		6.6(+)	8.3(+)	5.1(+)
1107	NADH-ubiquinone oxidoreductase 23 kDa	O00217		4.9(+)	5.2(+)	
1608	ATP synthase beta chain, mitochondrial precursor	P06576	5.0(+)	3.1(+)		2.8(+)
1610	Vimentin	P08670	5.5(+)	2.6(+)		
1705	Rab GDP dissociation inhibitor alpha	P31150	9.4(+)	5.8(+)		
2008	Enhancer of rudimentary homolog	Q14259		5.0(+)	3.7(+)	
2310	F-actin capping protein beta subunit	P47756	2.7(+)	2.2(+)	6.5(+)	3.0(+)
2310	Splice isoform 2 of F-actin capping protein beta subunit	P47756-2 isoform	2.7(+)	2.2(+)	6.5(+)	3.0(+)
2416	Zinc finger protein 266	Q14584	3.4(+)	2.4(+)	7.3(+)	2.8(+)
2419	Guanine nucleoide-binding protein G(O), alpha subunit 1	P09471	7.3(+)	2.1(+)	9.1(+)	9.5(+)
2419	Guanine nucleotide-binding protein G(O), alpha subunit 2	P29777	7.3(+)	2.1(+)	9.1(+)	9.5(+)
3013	Acyl-CoA-binding protein	P07108		2.7(+)	4.3(+)	
3222	Chain 1: Endoplasmic reticulum protein ERP29	P30040			4.1(+)	
4012	Cytochrome c oxidase polypeptide VIb	P14854		4.4(+)	8.7(+)	
4313	Aldose reductase	P15121	5.7(+)	9.6(+)	14.6(+)	8.1(+)
4616	Tryptophan 5-hydroxylase (Neuronal tryptophan hydroxylase)	Q8IWU9	10.6(+)	2.1(+)	7.8(+)	8.3(+)
5207	Glutathione S-transferase Mu-2	P28161	2.5(+)	2.8(+)	4.2(+)	2.9(+)
5617	Proto-oncogene tyrosine-protein kinase FYN	P06241	2.8(+)		3.9(+)	
6115	Neuronal protein NP25	Q9UI15		4.9(+)	4.6(+)	
6508	Isocitrate dehydrogenase [NADP] cytoplasmic	O75874	6.1(+)	6.7(+)	8.7(+)	4.4(+)
7503	Peroxisomal acyl-coenzyme A thioester hydrolase 2a	P49753	2.8(+)	2.0(+)	2.2(+)	2.5(+)
7519	Chain 1: matrix metalloproteinase-19	Q99542				3.1(+)
7106	40 kDa peptidyl-prolyl cis-trans isomerase	Q08752	4.3(+)		5.0(+)	5.1(+)

LH-NFPA = NFPA that expressed leuteinizing hormone, or lutropin; FSH-NFPA = NFPA that expressed follicle-stimulating hormone, or follitropin; LH/FSH-NFPA = NFPA that expressed both follicle-stimulating hormone and leuteinizing hormone; NF-NFPA = NFPA that had negative immunohistochemical stains for ACTH, FSH, GH, LH, prolactin, and TSH. Each adenoma was graded blindly by a neuropathologist from 0–4 for intensity of staining for each peptide hormone. (−) = decreased relative to controls; (−−) = lost relative to controls; (+) = increased relative to controls; (+/−) = no change relative to controls.

### Comparative analysis of DEP profiles derived from four NFPA subtypes relative to controls

An overlapping analysis (Figure 3) among 59 DEPs in NF-NFPA, 65 DEPs in LH-NFPA, 63 DEPs in FSH-NFPA, and 55 DEPs in LH/FSH-NFPA relative to controls (Table 1) revealed that 44 DEPs were common to four NFPA subtypes (NF-, LH-, FSH-, and LH/FSH-). Those 44 common DEP-spots among four NFPA subtypes (NF-, LH-, FSH-, and LH/FSH-) include ten upregulated and 34 downregulated DEPs. Those ten common upreguated DEPs were F-actin capping protein beta unit, splice isoform 2 of F-actin capping protein beta unit, zinc finger protein 266, G(O)-protein alpha subunit 1, G(O)-protein alpha subunit 2, aldose reductase, tryptophan 5-hydroxylase, glutathione S-transferase Mu-2, isocitrate dehydrogenase [NADP] cytoplasmic, and peroxisomal acyl-coenzyme A thioester hydrolase 2A. The 34 common down-regulated DEPs were somatotropin chain 1, splice isoform 2 of somatotropin precursor, growth hormone variant precursor, prolactin chain 1, splice isoform IL15-S21AA of interleukin-15 precursor, factor X light chain, cytokeratin 16, alpha crystalline C chain, 14-3-3 protein tau, secretagogin, Mu-crystallin homolog, MIMECAN chain 1, tissue transglutaminase, ATP binding protein associated with cell differentiation, N6-adenosine-methyltransferase 70 kDa subunit, hemoglobin beta unit variant, hemoglobin beta-2 chain, and insulin-like growth factor binding. Among them, 16 common DEP-spots contained somatotropin and its isoforms with different p*I* and M_*r*_, and four common DEPs contained prolactin and its isoforms with different p*I* and M_*r*_. Those common DEPs hint at the common molecular mechanisms and signaling pathways involved in four NFPA subtypes.

Four DEPs (SSP 2101, 4215, 2624, and 1608) were common to only three NFPA subtypes (NF-, LH-, and LH/FSH-), including three down-regulated proteins (somatotropin chain 1, prolactin precursor, and dipeptidyl-peptidase II) and one up-regulated protein (mitochondrial ATP synthase beta chain). Three DEP-spots (SSP 4216, 8102, and 1105) were common to only three NFPA subtypes (LH-, FSH-, and LH/FSH-), including two down-regulated proteins (prolactin chain 1, and phospholipid hydroperoxide glutathione peroxidase) and one up-regulated protein (lactoylglutathione lyase). Two DEP-spots (SSP 2001, and 7106) were common to only three NFPA subtypes (NF-, FSH-, and LH/FSH-), including one down-regulated protein (CD59 glycoprotein) and one up-regulated protein (40 kDa peptidyl-prolyl cis-trans isomerase).

Eight DEPs (SSP 4921, 8208, 0118, 1107, 2008, 3013, 4012, and 6115) were common to only two NFPA subtypes (LH-, and FSH-), including two down-regulated proteins (collagen alpha 2 VI chain, and Ig kappa chain V-III region SIE) and six up-regulated proteins (lactoylglutathione lyase, NADH-ubiquinone oxidoreductase 23 kDa, enhancer of rudimentary homolog, acyl-CoA-binding protein, cytochrome c oxidase polypeptide VIb, and neuronal protein NP25). Four DEPs (SSP 0812, 5003, 1610, and 1705) were common to only two NFPA subtypes (NF-, and LH-), including two down-regulated proteins (tumor rejection antigen 1, and hemoglobin beta-2 chain) and two up-regulated proteins (vimentin, and Rab GDP dissociation). Three DEPs (SSP 2303, 3210, and 5617) were common to only two NFPA subtypes (NF-, and FSH-), including two down-regulated proteins (serine/threonine protein phosphatase 2A - 55 kDa regulatory subunit B alpha isoform, and heat shock protein 27) and one up-regulated protein (proto-oncogene tyrosine-protein kinase FYN). One DEP-spot (SSP 7016) was common to only two NFPA subtypes (FSH-, and LH/FSH-), which was a down-regulated protein (hemoglobin alpha-2 chain).

Two DEPs (SSP 1012, and 1117) were specific to NF-NFPAs, which were two down-regulated proteins (splice isoform 3 of alpha-s1 casein precursor, and apolipoprotein A-I). Two DEPs (SSP 2311, and 3222) were specific to FSH-NFPAs, including one down-regulated protein (L-Myc-1 protooncogene protein) and one up-regulated protein (endoplasmic reticulum protein ERP29). One DEP-spot (SSP 1017) was specific to LH-NFPAs, which contained two down-regulated proteins (somatotropin, and splice isoform 2 of somatotropin precursor). One DEP-spot (SSP 7519) was specific to LH/FSH-NFPAs, which was one up-regulated protein (matrix metalloproteinase-19).

### Variations in signaling pathway networks among four NFPA subtypes

Each protein functions in an organized, systematic, and dynamic pathway network system. The Ingenuity Pathway Analysis (IPA) program was used to clarify pathway networks that involve each DEP and to address potential biological functions of those DEPs in each NFPA subtype (NF-, LH-, FSH-, and LH/FSH-). DEPs were analyzed with IPA to determine significant pathway networks, canonical pathways, disease biological events, and biological toxicity events and to reveal variations in signaling pathway networks to provide pathway networks, canonical pathways, disease biological events, and biological toxicity events that were common or specific to each NFPA subtype.

### Differences in pathway networks

**Figure 4:**
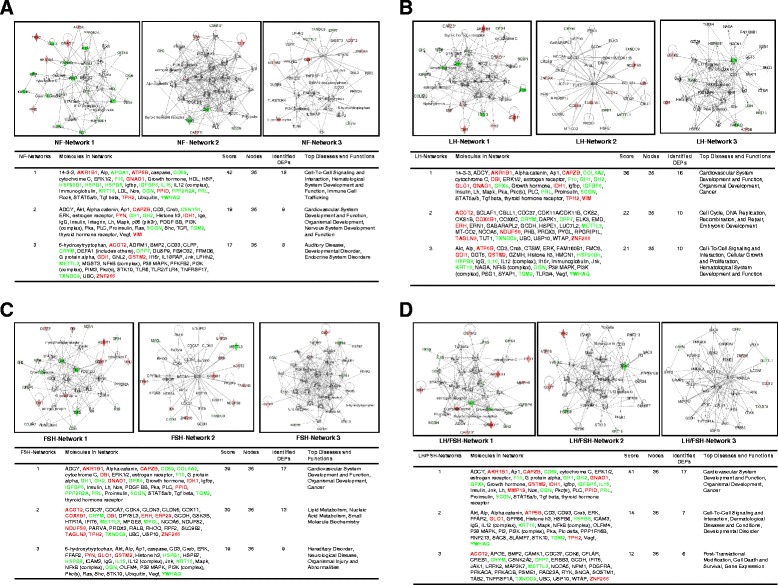
**Signal pathway networks in different subtypes of clinically NFPAs. A**. NF-NFPA. **B**. LH-NFPA. **C**. FSH-NFPA. **D**. LH/FSH-NFPA.

For NF-NFPAs, pathway network analysis of 59 DEPs in NF-NFPAs identified three statistically significant pathway networks that involve NF-NFPA-related DEPs (Figure 4A). Those nodes in the top-half of Figure 4A correspond to molecules (genes; proteins) summarized in the bottom-half of Figure 4A. NF-network 1 functions in cell-to-cell signaling and interaction, hematological system development and function, and immune cell tracking; and includes 35 nodes (genes; proteins). Among those 35 nodes, 18 DEPs (51% of total nodes) were MS-identified. ERK1/2, PRL, APOA1, TGF beta, LDL, NOS, IL12 complex, HSPB1, and YWHAQ play key roles in this network. NF-network 2 functions in cardiovascular system development and function, organism development, and nervous system development and function; and includes 35 nodes (genes; proteins). Among those 35 nodes, nine DEPs (26% of total nodes) were MS-identified. ERK, GH1, PKA, RAS, Akt, VEGF, MAPK, FYN, p85 (pik3r), PI3K complex, and insulin play key roles in this network. NF-network 3 functions in auditory disease, development disorder, and endocrine system disorders; and includes 35 nodes (genes; proteins). Among those 35 nodes, eight DEPs (23% of total nodes) were MS-identified. UBC, FBXO32, P38 MAPK, NFkB complex, PI3K complex, and PKC play key roles in this network.

For LH-NFPAs, pathway network analysis of 65 DEPs in LH-NFPAs identified three statistically significant pathway networks that involve LH-NFPA-related DEPs (Figure 4B). Those nodes in the top-half of Figure 4B corresponded to molecules (genes; proteins) summarized in the bottom-half of Figure 4B. LH-network 1 functions in cardiovascular system development and function, organism development, and cancer; and includes 35 nodes (genes; proteins). Among those 35 nodes, 16 DEPs (46% of total nodes) were MS-identified. ERK1/2, PRL, GH1, LH, MAPK, PKC, PKA, 14-3-3, estrogen receptor, TGF beta, and insulin play key roles in this network. LH-network 2 functions in cell cycle, DNA replication, recombination, and repair, embryonic development; and includes 35 nodes (genes; proteins). Among those 35 nodes, 10 DEPs (29% of total nodes) were MS-identified. UBC, CDC37, and COX6B1 play key roles in this network. LH-network 3 functions in cell-to-cell signaling and interaction, cellular growth and proliferation, hematological system development and function; and includes 35 nodes (genes; proteins). Among those 35 nodes, 10 DEPs (29% of total nodes) were MS-identified. P38 MAPK, Akt, ERK, NF-kB complex, CD3, IgG, IL15, IL12 complex, Jnk, VEGF, and TGM2 play key roles in this network.

For FSH-NFPAs, pathway network analysis of 63 DEPs in FSH-NFPAs identified three statistically significant pathway networks that involve FSH-NFPA-related DEPs (Figure 4C). Those nodes in the top-half of Figure 4C corresponded to molecules (genes; proteins) that were summarized in the bottom-half of Figure 4C. FSH-network 1 functions in cardiovascular system development and function, organism development, and cancer; and includes 35 nodes (genes; proteins). Among those 35 nodes, 17 DEPs (49% of total nodes) were MS-identified. ERK1/2, PKA, PLC, TGF beta, TGM2, LH, GH1, PRL, and insulin play key roles in this network. FSH-network 2 functions in lipid metabolism, nucleic acid metabolism, and small-molecule biochemistry; and includes 35 nodes (genes; proteins). Among those 35 nodes, 13 DEPs (37% of total nodes) were MS-identified. UBC, CDC37, and GSK3B play key roles in this network. FSH-network 3 functions in hereditary disorder, neurological disease, organismal injury and abnormalities; and includes 35 nodes (genes; proteins). Among those 35 nodes, 9 DEPs (26% of total nodes) were MS-identified. P38 MAPK, Akt, ERK, NF-kB complex, VEGF, Jnk, MAPK, PI3K complex, Creb, PKC, and HSPB play key roles in this network.

For LH/FSH-NFPAs, pathway network analysis of 55 DEPs in LH/FSH-NFPAs identified three statistically significant pathway networks that involve LH/FSH-NFPA-related DEPs (Figure 4D). Those nodes in the top-half of Figure 4D corresponded to those molecules (genes; proteins) summarized in the bottom-half of Figure 4D. LH/FSH-network 1 functions in cardiovascular system development and function, organism development, and cancer; and includes 35 nodes (genes; proteins). Among those 35 nodes, 17 DEPs (49% of total nodes) were MS-identified. ERK1/2, Jnk, AP1, NOS, AKR1B1, PRL, LH, PLC, PKC, GH1, STAT5a/b, estrogen receptor, ADCY, and insulin play key roles in this network. LH/FSH-network 2 functions in cell-to-cell signaling and interaction, dermatological diseases and conditions, and development disorders; and includes 35 nodes (genes; proteins). Among those 35 nodes, 7 DEPs (20% of total nodes) were MS-identified. MAPK, TGM2, ERK, NF-kB complex, IL12 complex, VEGF, Creb, Akt, PKA, P38 MAPK, IgG, and CD3 play key roles in this network. LH/FSH-network 3 functions in post-translational modification, cell death and survival, and gene expression; and includes 35 nodes (genes; proteins). Among those 35 nodes, 6 DEPs (17% of total nodes) were MS-identified. UBC, CDC37, SOSTM1, PRKACA, and CREB1 play key roles in this network.

Comprehensive analysis of four sets of pathway networks (Figure 4A-D) revealed: (i) The pathway network (NF-network 2, LH-network 1, FSH-network 1, and LH/FSH-network 1) was highly similar among NF-, LH-, FSH-, and LH/FSH-NFPAs, and functions in cancer, cardiovascular system development and function, organism development, and nervous system development and function; and nodes ERK1/2, GH1, LH, insulin, and PRL play key roles in this common pathway network. However, they are not the same, and display differences among NF-, LH-, FSH- and LH/FSH-NFPAs. (ii) The pathway network (NF-network 1, LH-network 3, and LH/FSH-network 2) is very similar among NF-, LH-, and LH/FSH-NFPAs, and functions in cell-to-cell signaling and interaction, hematological system development and function, immune cell tracfficking, cell growth and proliferation, dermatological diseases and conditions, and developmental disorder; network FSH-network 3 is very similar to the above-mentioned three networks, and functions in hereditary disorder, neurological disease, and organism injury and abnormalities. (iii) The network NF-network 3, LH-network 2, FSH-network 2, and LH/FSH-network 3 are basically different, however, they have common nodes UBC and CDC37, and the other nodes are almost different.

### Difference in canonical pathways

**Figure 5:**
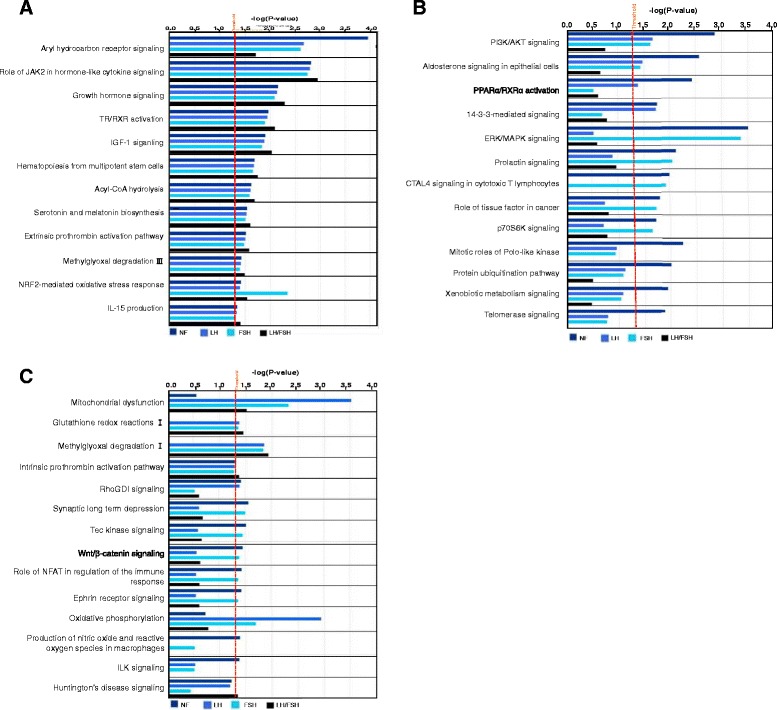
**Canonical pathways that are common and specific to different subtypes of clinically NFPAs (Panels A, B, and C).** The blue bar means NF-NFPA. The light blue bar means LH-NFPA. The green bar means FSH-NFPA. The dark bar means LH/FSH-NFPA.

Among those DEPs in four NFPA subtypes, pathway network analysis identified 34 statistically significant canonical pathways that involve DEPs in NF-NFPAs, 21 in LH-NFPAs, 28 in FSH-NFPAs, and 18 in LH/FSH-NFPAs. A comparative analysis of those significant canonical pathways was performed among NF-, LH-, FSH-, and LH/FSH-NFPAs (Figure 5). (i) A total of 12 canonical pathways was common to four NFPA subtypes, including aryl hydrocarbon receptor signaling, role of JAK2 in hormone-like cytokine signaling, growth hormone signaling, TR/RXR activation, IGF-1 signaling, hematopoiesis from multipotent stem cells, acyl-CoA hydrolysis, serotonin and melatonin biosynthesis, extrinsic prothrombin activation pathway, methylglyoxal degradation III, NRF2-mediated oxidative stress response, and IL-15 production (Figure 5A). (ii) Two significant canonical pathways were common to only three NFPA subtypes (NF-, LH-, and FSH-), including PI3K/AKT signaling, and aldosterone signaling in epithelial cells (Figure 5B). (iii) Three significant canonical pathways were common to only three NFPA subtypes (LH-, FSH-, and LH/FSH-), including mitochondrial dysfunction, glutathione redox reactions I, and methylglyoxal degradation I (Figure 5C). (iv) One significant canonical pathway, intrinsic prothrombin activation pathway, was common to only three NFPA subtypes (NF-, LH-, and LH/FSH-) (Figure 5C). (v) Three significant canonical pathways were common to only two NFPA subtypes (NF-, and LH-), including PPARα/RXRα activation, 14-3-3-mediated signaling, and RhoGDI signaling (Figure 5B and C). (vi) Ten significant canonical pathways were common to only two NFPA subtypes (NF-, and FSH-), including ERK/MAPK signaling, prolactin signaling, CTAL4 signaling in cytotoxic T lymphocytes, role of tissue factor in cancer, p70S6K signaling, synaptic long term depression, Tec kinase signaling, Wnt/β-catenin signaling, role of NFAT in regulation of the immune response, and Ephrin receptor signaling (Figure 5B and C). (vii) One significant canonical pathway, oxidative phosphorylation, was common to only two NFPA subtypes (LH-, and FSH-) (Figure 5C). (viii) Six significant canonical pathways were specific to only NF-NFPAs, including mitotic roles of Polo-like kinase, protein ubiquitination pathway, xenobiotic metabolism signaling, telomerase signaling, production of nitric oxide and reactive oxygen species in macrophages, and ILK signaling (Figure 5B and C). (ix) One significant canonical pathway, Huntington’s disease signaling, was specific to only LH/FSH-NFPAs (Figure 5C).

### Differences in disease biological events

Among DEPs in four NFPA subtypes, pathway network analysis identified 77 statistically significant disease biological events that involve in NF-NFPAs, 76 in LH-NFPAs, 76 in FSH-NFPAs, and 79 in LH/FSH-NFPAs. A comparative analysis of those significant disease biological events was performed among NF-, LH-, FSH-, and LH/FSH-NFPAs (Additional file 1: Figure S1). (i) A total of 70 disease biological functional events were common to four NFPA subtypes (NF-, LH-, FSH-, and LH/FSH-) (Additional file 1: Figure S1A-E), including cancer, development and function of cellular, tissue, and multiple organ systems, morphology of cell, tissue, organ, and tumor, multiple diseases and disorders including inflammatory and neurological diseases, inflammatory and immune responses, cell-to-cell signaling and interaction, cell death and survival, cellular movement, cellular growth and proliferation, protein synthesis, lipid metabolism, molecular transport, cell cycle, carbohydrate metabolism, energy production, vitamin and mineral metabolism, nucleic acid metabolism, DNA replication recombination and repair, amino acid metabolism, drug metabolism, post-translational modification, and behaviors, etc. (ii) Some disease biological events were not common to all NFPA subtypes (Additional file 1: Figure S1F), including protein degradation, protein folding, protein trafficking, and free radical scavenging, etc.

### Difference in biological toxicity events

Among DEPs in four NFPA subtypes, pathway network analysis identified 21 statistically significant biological toxicity events that involve DEPs in NF-NFPAs, 12 in LH-NFPAs, 14 in FSH-NFPAs, and 17 in LH/FSH-NFPAs. A comparative analysis of those significant biological toxicity events was performed among NF-, LH-, FSH-, and LH/FSH-NFPAs (Additional file 1: Figure S2). (i) Ten biological toxicity events were common to four NFPA subtypes (NF-, LH-, FSH-, and LH/FSH-), including cardiac arrhythmia, liver hypertrophy, renal inflammation, renal nephritis, cardiac pulmonary embolism, cardiac stenosis, increased levels of bilirubin, kidney failure, increased levels of potassium, and glutathione depletion in liver. (ii) Two biological toxicity events were common to only three NFPA subtypes (NF-, FSH-, and LH/FSH-), including increased levels of hematocrit, and increased levels of red blood cells. (iii) Two biological toxicity events were common to only two NFPA subtypes (NF-, and LH/FSH-), including cardiac infraction, and cardiac necrosis/cell death. (iv) One biological toxicity event, liver hyperplasia/hyperproliferation, was common to only two NFPA subtypes (NF-, and LH-). (v) Two biological toxicity events were common to only two NFPA subtypes (NF-, and FSH-), including renal necrosis/cell death, and glomerular injury. (vi) Four biological toxicity events were specific to NF-NFPAs, including cardiac fibrosis, cardiac inflammation, cardiac arteriopathy, and liver cirrhosis. (vii) Three biological toxicity events were specific to LH/FSH-NFPAs, including heart failure, liver necrosis/cell death, and liver damage.

## Discussion

Reliability of DEP data is the most-important aspect of 2DGE-based comparative proteomics and for pathway network analysis. In order to achieve reliable DEP data of each NFPA subtype relative to controls, four strategies were applied: (a) spatial and quantitative reproducibility and linear dynamic range of 2DGE analysis systems were optimized and evaluated carefully [32,33]. An appropriate amount (70 μg) of protein for each 2DGE gel was used, and a 3-fold “cut-off” with statistical significance (p < 0.05) was used to determine a DEP. (b) For biological reproducibility, eight tissue samples for controls and three tissue samples for each NFPA subtype were used; and for technique reproducibility, each biological sample was analyzed with 2DGE for 3–5 times. Thus, a total of 30 gel images for controls and 9 gel images for each NFPA subtype was used to detect the DEPs with 3-fold cut-off value (p < 0.05) between each NFPA subtype relative to controls (see Figure 2). (c) proteomic heterogeneity of those eight controls was analyzed [34] and used to assist in the determination of DEPs for each NFPA subtype. (d) DEP data were correlated with comparative transcriptomics data, and validated with reverse transcriptase real-time polymerase chain reaction (RT-PCR) [15,35] to validate the DEP data.

In our long-term NFPA proteomics program, we demonstrated DEP profile between NFPAs and controls [15,35], including 50 DEPs (21 up-regulated and 29 down-regulated) contained in 72 2D gel-spots. Those DEPs also correlated with differentially expressed genes (DEGs; n = 284) from transcriptomics analysis, and were validated with RT-PCR [15,35]. Moreover, pathway network analysis of those 50 DEPs [15,35], 111 protein mapping data [11], and 17 nitroproteins [17-19] revealed four important signal pathway networks, including mitochondrial dysfunction, oxidative stress, cell-cycle dysregulation, and MAPK-signaling system abnormality [23]. However, these proteomic studies [15,35] and pathway network analysis [23] did not consider the NFPA heterogeneity, which is an important clinical problem with NFPAs. The differential expressions (NF-, LH-, FSH-, and LH/FSH-positive) of LH and FSH in NFPAs are the common NFPA subtypes.

The present study, for the first time, revealed proteome heterogeneity of four NFPA subtypes (NF-, LH-, FSH-, and LH/FSH-positive). A set of DEP and signaling pathway network data was achieved among four NFPA subtypes. A comprehensive analysis of those complicated DEP data, pathway networks, canonical pathways, disease biological events, and biological toxicity events revealed several signaling pathway systems that were common and specific to different NFPA subtypes and functioned in an NFPA, including MAPK-signaling abnormality, oxidative stress, mitochondrial dysfunction, and cell-cycle dysregulation.

### MAPK-signaling abnormality

This common signaling pathway network abnormality occurs among four NFPA subtypes. The biological significance of MAPK-signaling abnormality in a pituitary adenoma has been described [23]. MAPK signaling pathway network involves stimulus (mitogens, cytokines, growth factors, and stress, etc.), G-protein (Cdc42, Ras, Rac, and Rho), MAPKKK (Raf, Tpl2, MEKK, MLK, TAK, ASK, and TAO), MAPKK (MEK), MAPK (ERK, JNK, and P38), and responses (proliferation, differentiation, apoptosis, and migration). ERKs, JNKs, and p38-MAPKs are the three main MAPK subfamilies. ERK 1/2 is activated by MEK1/2, which is activated by Raf, Ras, and growth factors or mitogens; Raf activity, as the main effecter of Ras, is suppressed by cyclic AMP-dependent kinase (PKA) in a normal cell. JNKs are activated by MEK4/7, and p38-MAPKs are activated by MEK3/4/6. The upstream signal of MEK3/4/6/7 is from Rac, Rho, cdc42, cytokines, or stresses. NF-κB, TNFα, and interleukin-1 regulate this pathway system. The details of MAPK signaling pathways in cancer were reviewed [36-38]. MAPK pathways are emerging as potential therapeutic targets for cancer, and development of inhibitors of MAPK pathways is important for cancer therapy. Pathway analysis of DEPs among four NFPA subtypes demonstrated that ERK1/2, ERK, MAPK, GH1, Ras, and NF-κB were the key nodes in their pathway networks (Figure 4A-D); and the role of JAK2 in hormone-like cytokine signaling, growth hormone signaling, and ERK/MAPK signaling were signaling pathway networks in adenomas. Even though MAPK-signaling abnormalities are a common pathway system that function in four NFPA subtypes, the MAPK-signaling system is very complicated and includes multiple components, and some differences are present in the MAPK signaling network system among four NFPA subtypes. For example, networks that involve the MAPK-signaling system (Figure 4) are not the same among four NFPA subtypes; and ERK/MAPK signaling of the MAPK-signaling system is statistically significant in only NF- and FSH-NFPAs (Figure 5B).

### Oxidative stress (OS)

OS is a common signaling pathway system among four NFPA subtypes. The biological significance of oxidative stress in pituitary adenomas has been described in detail [23]. Many studies indicated the presence of nitric oxide synthase (NOS) in the human and rat pituitaries [39-43], and the increased NOS activities and its increased mRNA have been found in pituitary adenomas relative to controls [43,44]. Nitric oxide (NO) is involved in the hypothalamic-pituitary-adrenocortical axis [45]. NO activates release of luteinizing hormone-releasing hormone (LHRH) and follicle-stimulating hormone-releasing hormone (FSHRH) from the hypothalamus, and of LH and FSH from the pituitary [46-48], stimulates or inhibits the secretion of PRL [49], regulates growth hormone (GH) secretion in the normal human pituitary and in acromegaly [50,51], and modulates GH secretion in a dose-dependent manner in GH adenomatous cells from human pituitary adenomas [52]. Our pathway analysis of DEPs clearly revealed a statistically significant NRF2-mediated oxidative stress response canonical pathway common to each NFPA subtype. However, some differences in the oxidative stress system were found; for example, oxidative phosphorylation was common to only LH- and FSH-NFPAs (Figure 5C); and production of NO and reactive oxygen species in macrophages was specific to LH-NFPAs (Figure 5C).

### Mitochondrial dysfunction

This common pathway system occurs among LH-, FSH-, and LH/FSH-NFPAs, except NF-NFPAs (Figure 5C). The biological significance of mitochondrial dysfunction in human pituitary adenoma was described [23]. Mitochondrial dysfunction was confirmed with a mitochondrial morphological change in a pituitary tumor, including an increased number of mitochondria, ultrastructurally abnormal mitochondria, large mitochondria, mitochondrial swelling, and characteristic vesicular mitochondria. Anti-mitochondrial staining showed intense and granular mitochondria, and electron microscopy showed swollen mitochondria in the cytoplasm with featured lamellar cristae in the spindle-cell oncocytoma of the adenohypophysis. Notable differences in the structure and function of mitochondria appeared between cancer and normal cells, and included differences in mtDNA sequence, molecular composition, and metabolic activity [53,54]. Mitochondria involve multiple metabolic functions that include oxidative phosphorylation - an energy-generating process that couples oxidation of respiratory substances to ATP synthesis, oxidative decarboxylation of pyruvate, the tricarboxylic acid cycle, fatty-acid oxidation, glycolysis, intracellular homeostasis of inorganic ions such as calcium and phosphate, and intracellular apoptosis [54]. This present study revealed mitochondria-related signaling pathways, including serotonin and melatonin biosynthesis, oxidative phosphorylation, energy metabolism, carbohydrate metabolism, and oxidative stress, that function in human pituitary adenoma cells (Figure 5 and Additional file 1: Figure S1C). Mitochondrial DEPs, including NADH-ubiquinone oxidoreductase 23 kDa and ATP synthase beta chain, were identified in NFPAs.

### Cell-cycle dysregulation

This dysregulation is a common pathway system among four NFPA subtypes. The biological significance of cell-cycle dysregulation in human pituitary adenomas was described [23] and reviewed [55-58]. The present study clearly demonstrated cell-cycle network (Figure 4B) and cell cycle regulation (Additional file 1: Figure S1F) in NFPAs. DEP data clearly demonstrated that an important cell-cycle regulator, 14-3-3 protein, was down-regulated (NF: lost; LH: lost; FSH: 6.4-fold; and LH/FSH: 15.5-fold) in four NFPA subtypes relative to controls (Table 1). Furthermore, our previous nitroproteomic data demonstrated that a nitrated proteasome could interfere with functions of the ubiquitin-proteasome system in cell-cycle regulation [23]. Also, the present study clearly demonstrated that ubiquitin C (UBC) was the key node among four NFPA subtypes (Figure 4) to suggest that oxidative/nitrative stress might also be involved in cell-cycle dysregulation in human pituitary adenomas.

In summary, although those pathway systems are common to four NFPA subtypes, one must note that those pathway network systems are, in fact, different among four NFPA subtypes. First, most nodes had different expression levels among four NFPA subtypes - with strong evidence of those DEP data in Table 1 and Figure 3. Second, those pathway networks were not completely the same among four NFPA subtypes - with evidence in Figures 4 and 5. Third, the main pituitary hormones, growth hormone and prolactin, clearly demonstrated multiple isoforms, and were differentially expressed in four NFPA subtypes relative to controls (Table 1). Those abnormal expressions of those pituitary hormones significantly functioned in pathway systems such as MAPK-signaling system described above (Figures 4 and 5), and were involved in pathophysiological processes. Therefore, those DEP profiles (Table 1 and Figure 3), the functional characteristics of those DEPs, and variations in signaling pathway systems (Figures 4 and 5) provide a heterogeneous profile of human NFPA proteomes. Such a heterogeneous database is essential for an in-depth understanding of NFPA-proteome heterogeneity to accurately clarify basic NFPA molecular mechanisms and to discoverer biomarkers for effective diagnosis, therapy, and prognosis to achieve personalized medicine practice for an NFPA.

## Conclusions

The present study used large-scale quantitative proteomics and systems biology strategies to clarify variations in proteomes and network systems among four NFPA subtypes and to elucidate NFPA-proteome heterogeneity. Results demonstrate common and specific DEP profiles and pathway networks among four NFPA subtypes and for the first time reveal NFPA-proteome heterogeneity. Those data provide a starting point to discover effective biomarkers and effective targets to achieve personalized medicine practice for an NFPA. Further experiments and more biological specimens are needed to investigate the biological significance of each altered pathway system in an NFPA biological system.

## Methods

### Pituitary adenomas, control tissues, and preparation of proteins

Differential gel spots that contained DEPs between different NFPA subtypes versus controls were derived from re-analysis of 2DGE images [15,35] that included eight whole control pituitary tissues and twelve NFPA tissues, and each sample had three to five replicate 2DGE images. Also, DEPs were validated with the proteomic variation data of eight controls [34]. Clinical information of pituitary adenoma and control tissue samples was collected in Table 2. Collection and management of those tissue samples were approved by the Institutional Review Board (IRB) of the University of Tennessee Health Science Center. The detailed sample collection procedure was described [35]. Each control pituitary tissue (0.45-0.70 g; n = 8) and each NFPA tissue (15–75 mg; n = 12) was homogenized individually, lyophilized, and protein content was quantified; proteins (70 μg) were used for a 2DGE analysis. The detailed procedure of protein sample preparation was described [11].

**Table 2:** **Clinical information of clinically nonfunctional pituitary adenoma and control pituitary samples**

**Groups**	**Sample ID**	**Sex/Age**	**Clinical information**	**Immunohistochemistry**
NF-NFPA	T164	M/35	Non-functional, visual loss, 3 × 3.5 × 4 cm. Partial hypopituitarism	Neg.
	T217	M/39	Non-functional	Neg.
	T219	M/68	Non-functional, 1.9 × 2.3 × 2.2 cm, invasion of the right cavernous sinus	Neg.
LH-NFPA	T208	F/47	Non-functional, 2 × 2 × 2 cm	LH 1-2+
	T204	M/47	Non-functional	LH 3+
	T237	F/40	Non-functional, right cavernous sinus extension	LH 2+
FSH-NFPA	T57	F/59	Non-functional, 2 × 3 cm	FSH 1+
	T89	M/62	Non-functional, 2 × 2.3 × 2.3 cm	FSH 2+
	T77	M/67	Non-functional, 2 × 2.2 × 2.4 cm, questionable cavernous sinus	FSH 2+
LH/FSH-NFPA	T65	F/54	Non-functional, 4 × 4 × 4 cm, cavernous sinus invasion	LH 2+, FSH 1+
	T138	M/60	Non-functional, 2.9 × 3.1 × 3.5 cm	LH 2+, FSH 2+
	T185	M/66	Non-functional, 2.8 ×, 2 × 2.4 cm. Bilateral cavernous sinus invasion	LH 2-3+, FSH 2-3+
Control pituitary (Con)	C2	M/27	Black, none	DNT
	C3	F/40	White, Multiple toxic compounds. Blood: HepBb (+), HepC (+), HIV (−)	DNT
	C4	M/45	White, Drowning. Blood alcohol = 3.1 g/L; no other drugs detected. Blood: HepB (+), HepC (+), HIV (−)	DNT
	C5	M/36	White, Multiple toxic materials. Blood alcohol = 0.5 g/L. Blood: HepB (+), HepC (−), HIV (−)	DNT
	C7	F/34	Black, Gunshot wound to chest. Blood alcohol = 0.3 g/L; no drugs. Blood: HepB (+), HepC (−), HIV (−)	DNT
	C8	F	White, 15 h gunshot wound to head. No drugs or alcohol. Blood: HepB (−), HepC (−), HIV (−)	DNT
	C9	M/55	White, 12 h gunshot wound to chest. No alcohol or drugs. Blood: HepB (−), HepC (−), HIV (−)	DNT
	C10	F/47	White, Smoke inhalation. No drugs or alcohol. Numerous amylacea present in brain. Early autolytic changes to brain. Blood: HepB (−), HepC (+), HIV (−)	DNT

Note: All those hormones (ACTH, LH, FSH, PRL, GH, and TSH) were immunohistochemisty-tested in each pituitary adenoma tissue. Neg. = Immunohistochemical stains for ACTH, LH, FSH, PRL, GH, and TSH were negative. LH+ = nonfunctional pituitary adenoma that expressed leuteinizing hormone, or lutropin; FSH+ = nonfunctional pituitary adenoma that expressed follicle-stimulating hormone, or follitropin; FSH+, LH+ = nonfunctional pituitary adenoma that expressed both follicle-stimulating hormone and leuteinizing hormone. Adenomas were graded blindly by a neuropathologist (from 0–4) for the intensity of staining for each peptide hormone. NFPA = nonfunctional pituitary adenoma. DNT = do not test.

### 2DGE and 2D gel image analysis

First-dimension, IEF, was carried out on a Mulitphor II instrument (GE Health) with 70 μg protein sample and precast IPG strips (pH 3–10 NL; 180 × 3 × 0.5 mm). After equilibration of IEF-separated proteins, second-dimension, SDS-PAGE, was carried out with a 12% PAGE resolving gel (190 × 205 × 1.0 mm) in a vertical PROTEAN plus Dodeca™ Cell (Bio-Rad) which can analyze up to 12 gels at a time. 2DGE-separated proteins were visualized with a modified silver-staining method. Silver-stained 2DGE gels were digitized and analyzed with a PDQuest system (Version 7.1.0; Bio-Rad). A matched analysis set that contained 30 gel images from 8 control pituitary samples, 9 gel images from 3 NF-NFPA samples, 9 gel images from 3 LH-NFPA samples, 9 gel images from 3 FSH-NFPA samples, and 9 gel images from 3 LH/FSH-NFPA samples (a control pituitary as master gel) used to compare each DEP of NF-, LH-, FSH-, and LH/FSH-NFPAs relative to controls, respectively. Comparative analyses were carried out with the mean normalized volume between each NFPA subtype and controls. The “cutoff point” value for a significant difference of a differential spot was a three-fold difference. The detailed 2DGE method and 2D gel image analysis were described [11].

### MALDI-TOF PMF and LC-ESI-MS/MS analyses

Each 2D gel-spot that contained a DEP was excised. Protein was subjected to in-gel digestion with trypsin, purification of tryptic peptides with a Zip-TipC18 micro-column, followed by analysis with a Perseptive Biosytems MALDI-TOF Voyager DE-RP mass spectrometer (Framingham, MA, USA) and with an LCQ^Deca^ mass spectrometer (LC-ESI-Q-IT) equipped with a standard electrospray source (ThermoFinnigan, San Jose, CA, USA). For MALDI-TOF MS, peptide mass fingerprint (PMF) data were generated; PMF data were used to identify protein with a search of the UniProt database with search software PeptIdent (http://us.expasy.org/tools/peptident.html) and Mascot (http://www.Matrixscience.com). For LC-ESI-Q-IT MS, tandem mass spectrometry (MS/MS) data were generated; MS/MS data were used to identify protein with a search of the UniProt and NCBInr databases with the SEQUEST software. Detailed experimental procedures were described [11].

### Pathway network analysis

The Ingenuity Pathway Analysis (IPA) system was used to obtain cellular pathways that might be modified by protein changes identified in these experiments. IPA automatically generated networks of gene, protein, small-molecule, drug, and disease associations on the basis of “hand-curated” data held in a proprietary database. Identifiers (Swiss-Prot identification number) of DEPs were uploaded as an Excel spreadsheet file into the Ingenuity software (Ingenuity Systems, Redwood City, CA, USA). Each human identification number was mapped to its corresponding molecule in the Ingenuity Pathway Knowledge Base. Biological functions assigned to each network were ranked according to significance of that biological function to the network. Protein networks were algorithmically generated based on their connectivity and were assigned a score. The score was used to rank networks according to how relevant they were to the proteins in the input data set. The network was presented as a graph that indicated the molecular relationship between proteins.

## Additional file

Additional file 1: Figure S1. Significant disease biological events that are common and specific to different subtypes of clinically NFPAs. The blue bar means NF-NFPA. The light blue bar means LH-NFPA. The green bar means FSH-NFPA. The dark bar means LH/FSH-NFPA. **Figure S2.** Significant biological toxicity events that are common and specific to different subtypes of clinically NFPAs. The blue bar means NF-NFPA. The light blue bar means LH-NFPA. The green bar means FSH-NFPA. The dark bar means LH/FSH-NFPA.

## Abbreviations

ACTH: Adrenocorticotropic hormone
CHAPS: 3-(3-cholamidopropyl)dimethylammonio-1-propanesulfonate
Con: Control pituitary
DEP: Differentially expressed protein
DTT: Dithiothreitol
ERK: Extracellular signal-regulated kinase
ESI: Electrospray ionization
FPA: Functional pituitary adenoma
FSH: Follicle-stimulating hormone
GH: Growth hormone
IEF: Isoelectric focusing
IPA: Ingenuity Pathway Analysis
IPG: Immobilized pH gradient
LC: Liquid chromatography
LH: Luteinizing hormone
MALDI: Matrix-assisted laser desorption ionization
MAPK: Mitogen activated protein kinase
MS/MS: Tandem mass spectrometry
NF: Negative hormone expression
NFPA: Nonfunctional pituitary adenoma
PMF: Peptide mass fingerprint
SDS-PAGE: Sodium dodecyl sulfate–polyacrylamide gel electrophoresis
2DGE: Two-dimensional gel electrophoresis
TEMED: Tetramethyl ethylenediamine
TOF: Time-of-flight
