# Supplementary material for: Heterogeneity analysis of the proteomes in clinically nonfunctional pituitary adenomas
Source: BMC Med Genomics. 2014 Dec 24;7:69. doi: 10.1186/s12920-014-0069-6 (PMC4302698; doi:10.1186/s12920-014-0069-6)
Supplement: Additional file 1: Figure S1. — Significant disease biological events that are common and specific to different subtypes of clinically NFPAs. The blue bar means NF-NFPA. The light blue bar means LH-NFPA. The green bar means FSH-NFPA. The dark bar means LH/FSH-NFPA. Figure S2. Significant biological toxicity events that are common and specific to different subtypes of clinically NFPAs. The blue bar means NF-NFPA. The light blue bar means LH-NFPA. The green bar means FSH-NFPA. The dark bar means LH/FSH-NFPA. [file 12920_2014_69_MOESM1_ESM.ppt]

## Slide 1
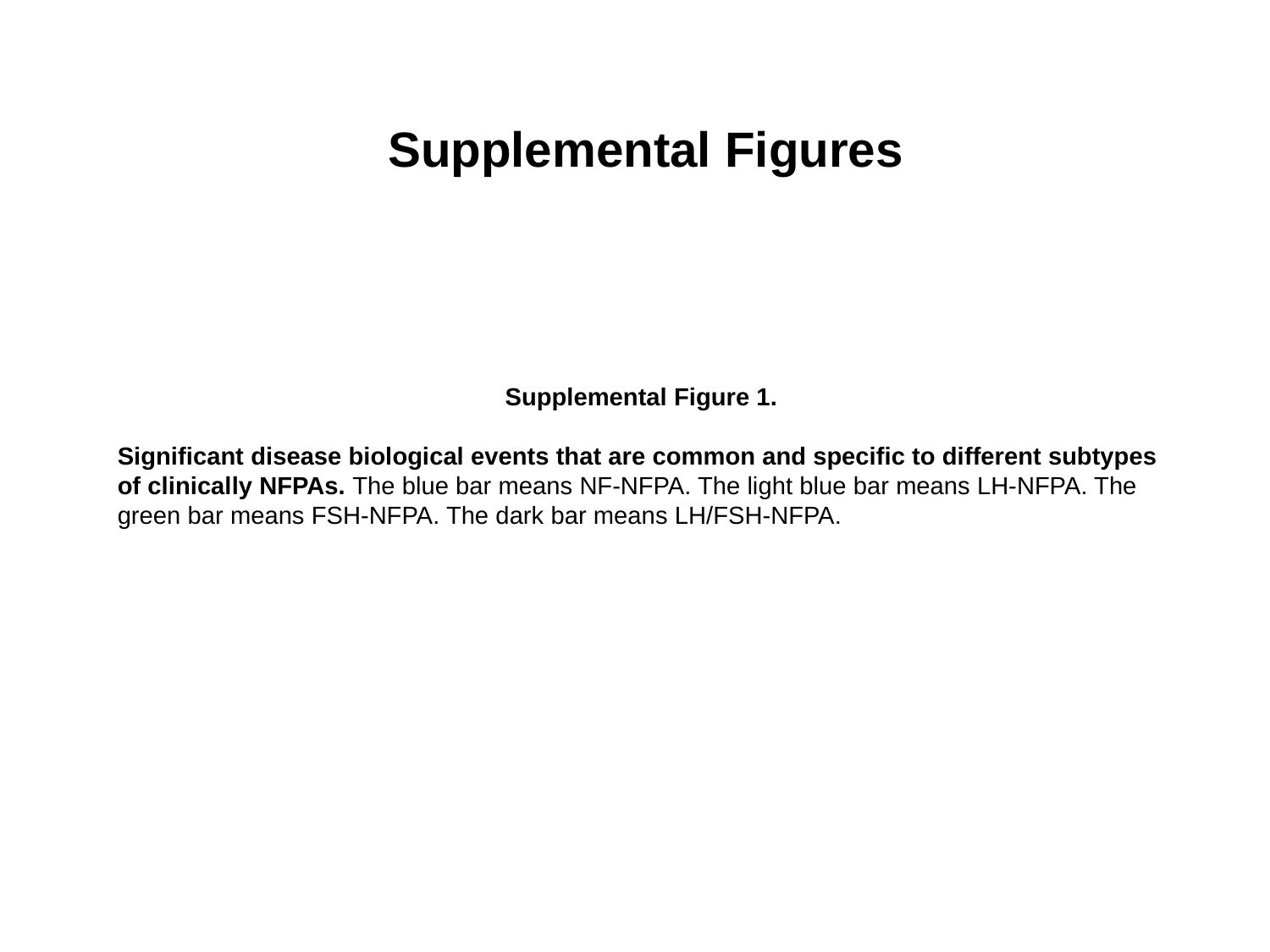

Supplemental Figures
Supplemental Figure 1.
Significant disease biological events that are common and specific to different subtypes of clinically NFPAs. The blue bar means NF-NFPA. The light blue bar means LH-NFPA. The green bar means FSH-NFPA. The dark bar means LH/FSH-NFPA.

## Slide 2
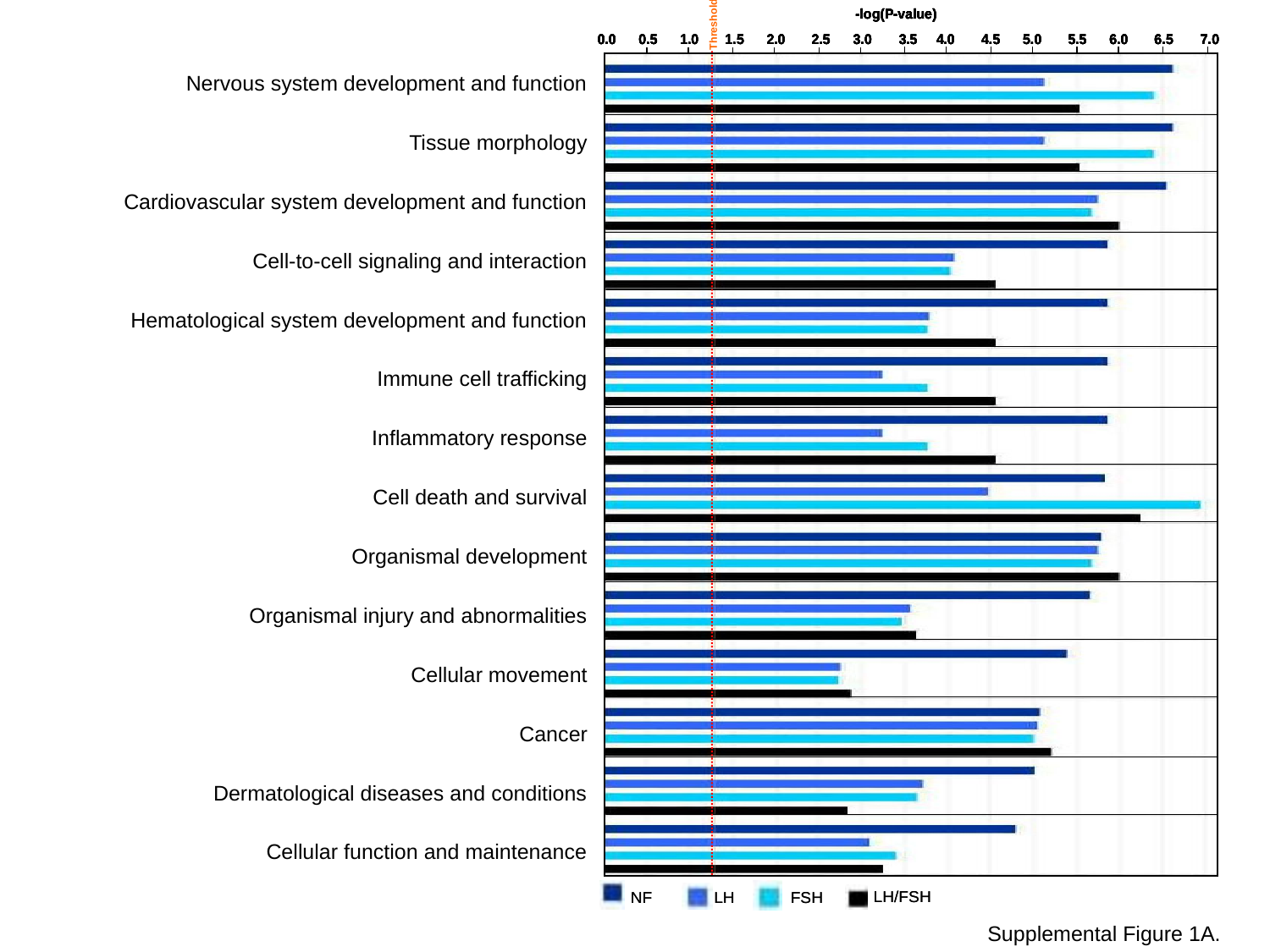

Threshold
Nervous system development and function
Tissue morphology
Cardiovascular system development and function
Cell-to-cell signaling and interaction
Hematological system development and function
Immune cell trafficking
Inflammatory response
Cell death and survival
Organismal development
Organismal injury and abnormalities
Cellular movement
Cancer
Dermatological diseases and conditions
Cellular function and maintenance
Supplemental Figure 1A.

## Slide 3
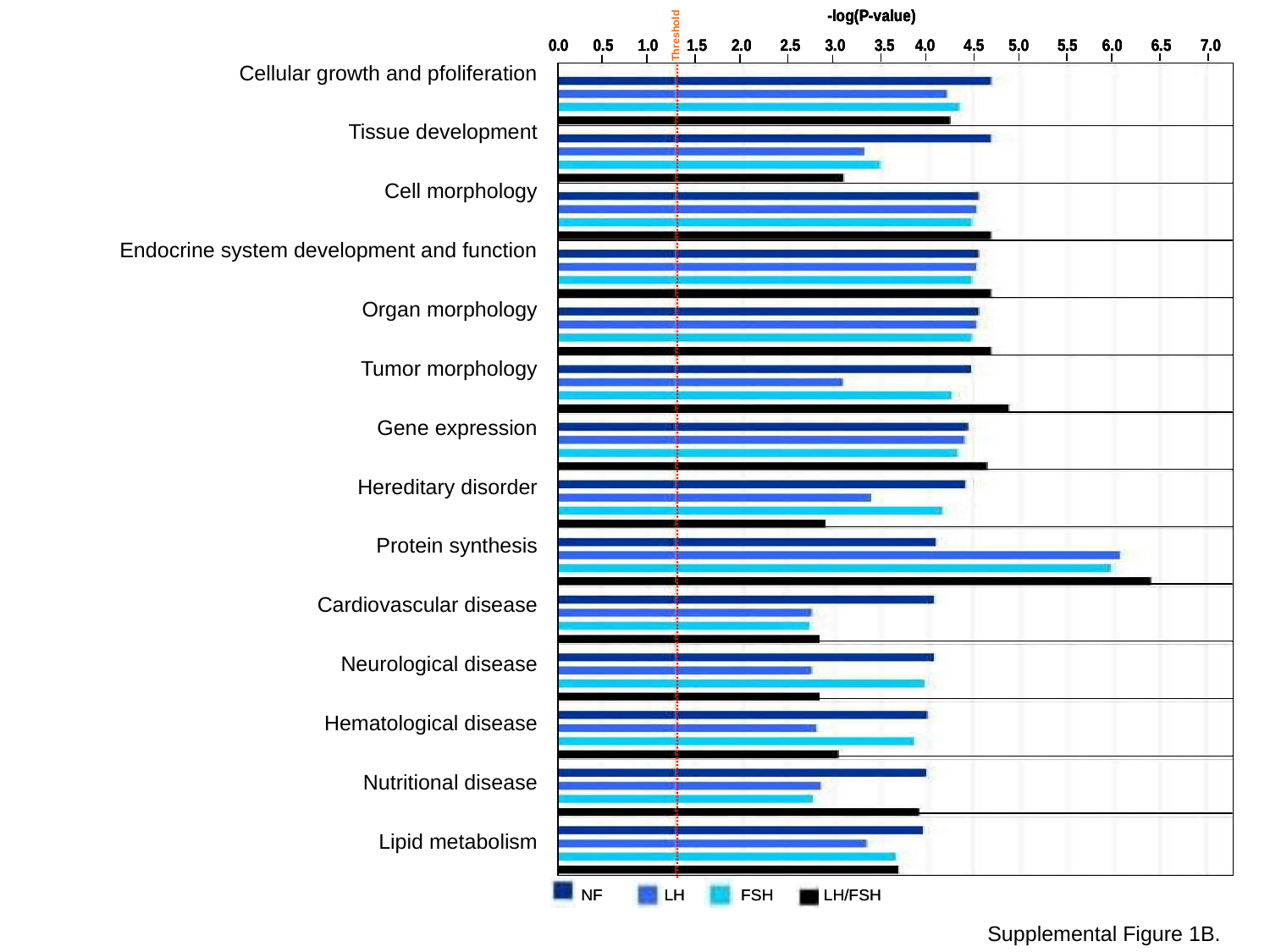

Threshold
Cellular growth and pfoliferation
Tissue development
Cell morphology
Endocrine system development and function
Organ morphology
Tumor morphology
Gene expression
Hereditary disorder
Protein synthesis
Cardiovascular disease
Neurological disease
Hematological disease
Nutritional disease
Lipid metabolism
Supplemental Figure 1B.

## Slide 4
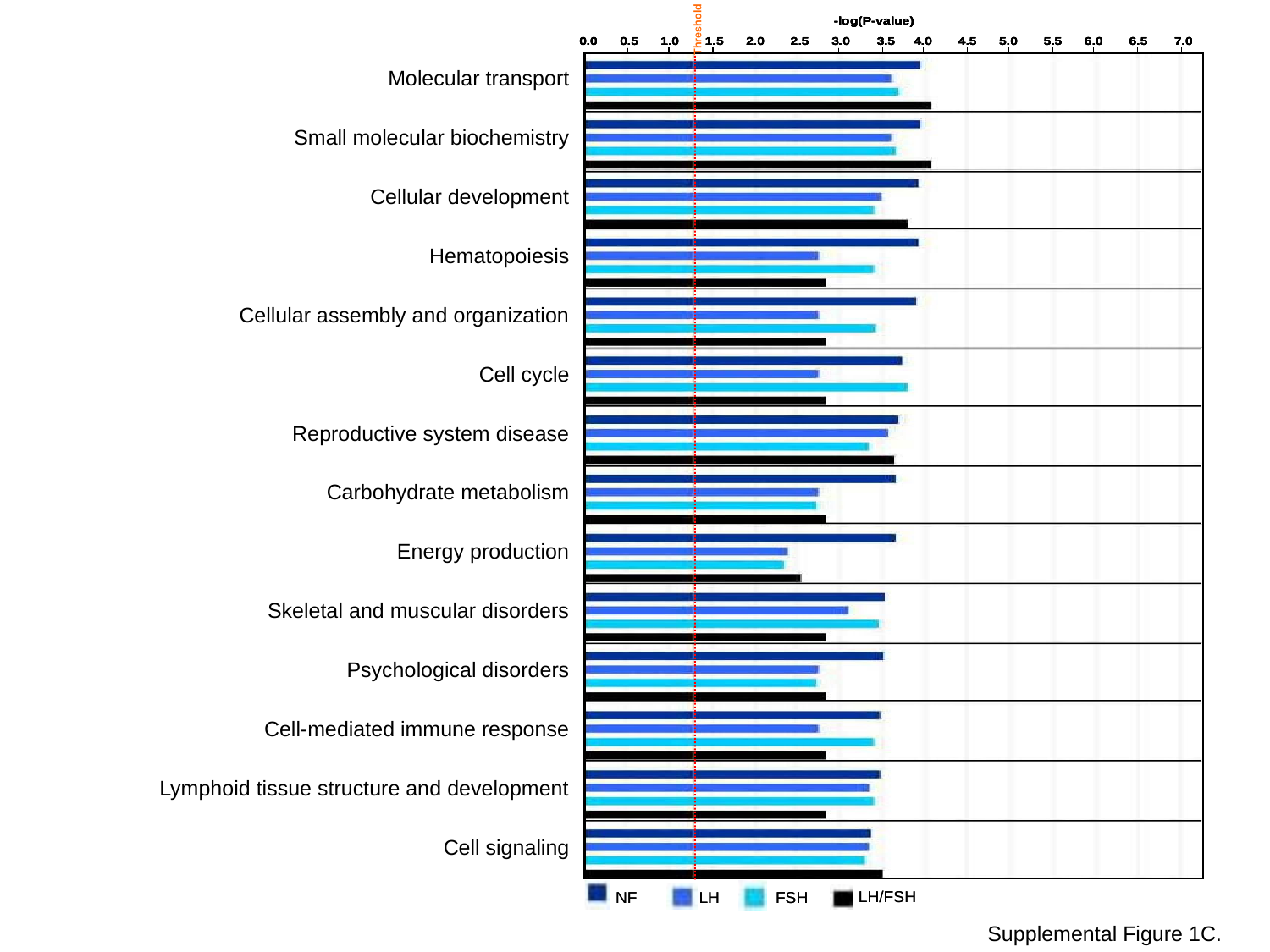

Threshold
Molecular transport
Small molecular biochemistry
Cellular development
Hematopoiesis
Cellular assembly and organization
Cell cycle
Reproductive system disease
Carbohydrate metabolism
Energy production
Skeletal and muscular disorders
Psychological disorders
Cell-mediated immune response
Lymphoid tissue structure and development
Cell signaling
Supplemental Figure 1C.

## Slide 5
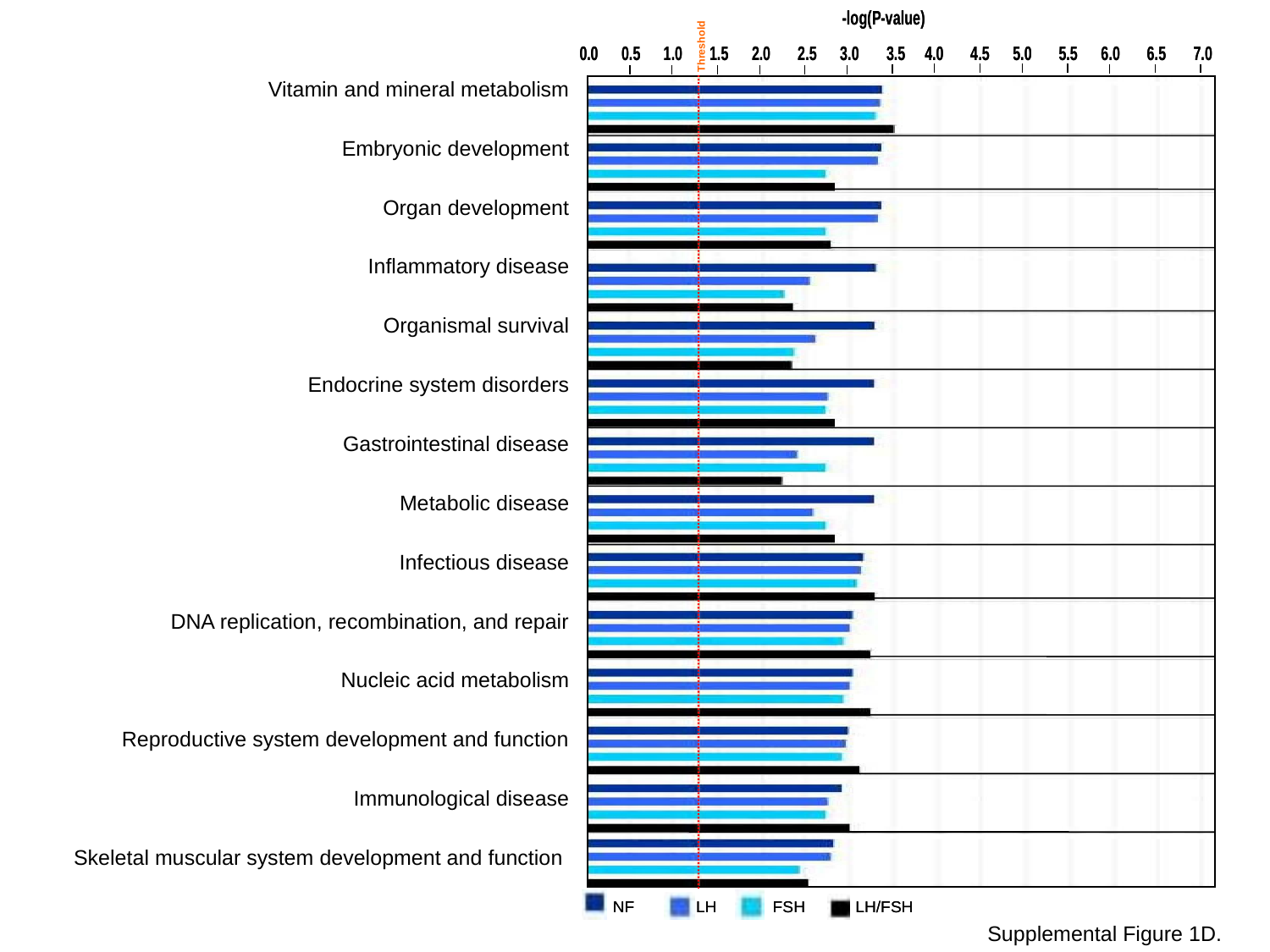

Threshold
Vitamin and mineral metabolism
Embryonic development
Organ development
Inflammatory disease
Organismal survival
Endocrine system disorders
Gastrointestinal disease
Metabolic disease
Infectious disease
DNA replication, recombination, and repair
Nucleic acid metabolism
Reproductive system development and function
Immunological disease
Skeletal muscular system development and function
Supplemental Figure 1D.

## Slide 6
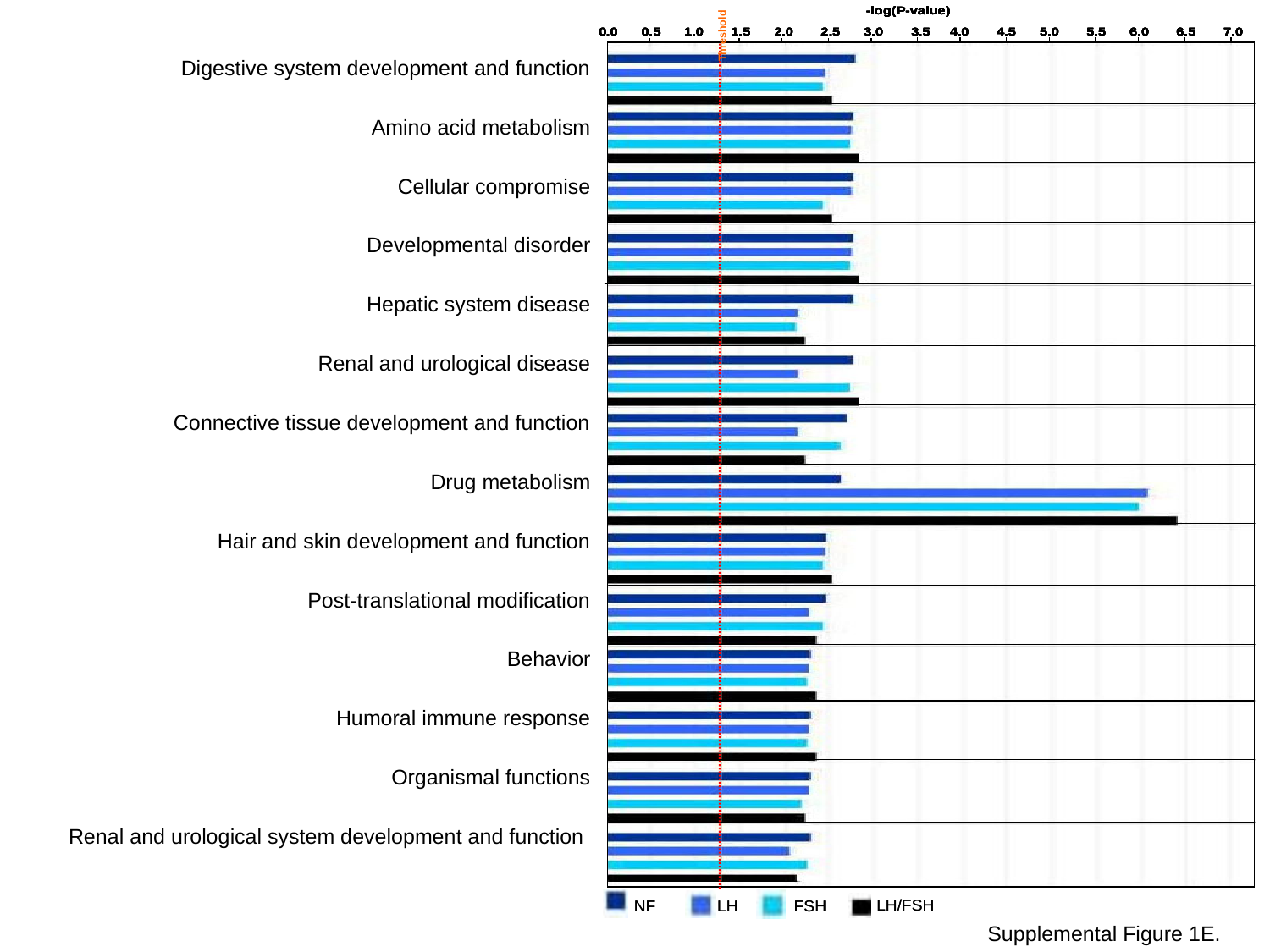

Threshold
Digestive system development and function
Amino acid metabolism
Cellular compromise
Developmental disorder
Hepatic system disease
Renal and urological disease
Connective tissue development and function
Drug metabolism
Hair and skin development and function
Post-translational modification
Behavior
Humoral immune response
Organismal functions
Renal and urological system development and function
Supplemental Figure 1E.

## Slide 7
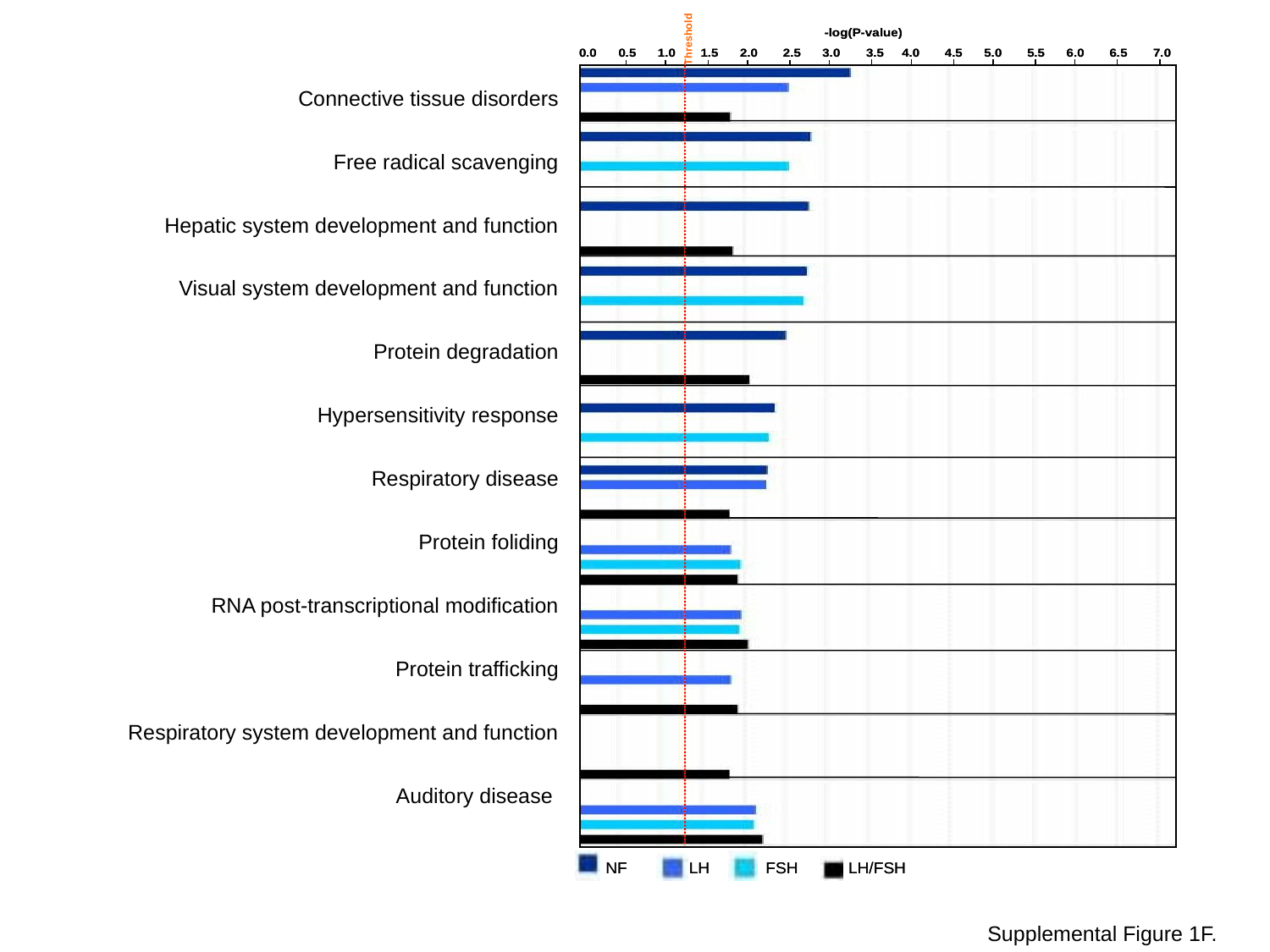

Threshold
Connective tissue disorders
Free radical scavenging
Hepatic system development and function
Visual system development and function
Protein degradation
Hypersensitivity response
Respiratory disease
Protein foliding
RNA post-transcriptional modification
Protein trafficking
Respiratory system development and function
Auditory disease
Supplemental Figure 1F.

## Slide 8
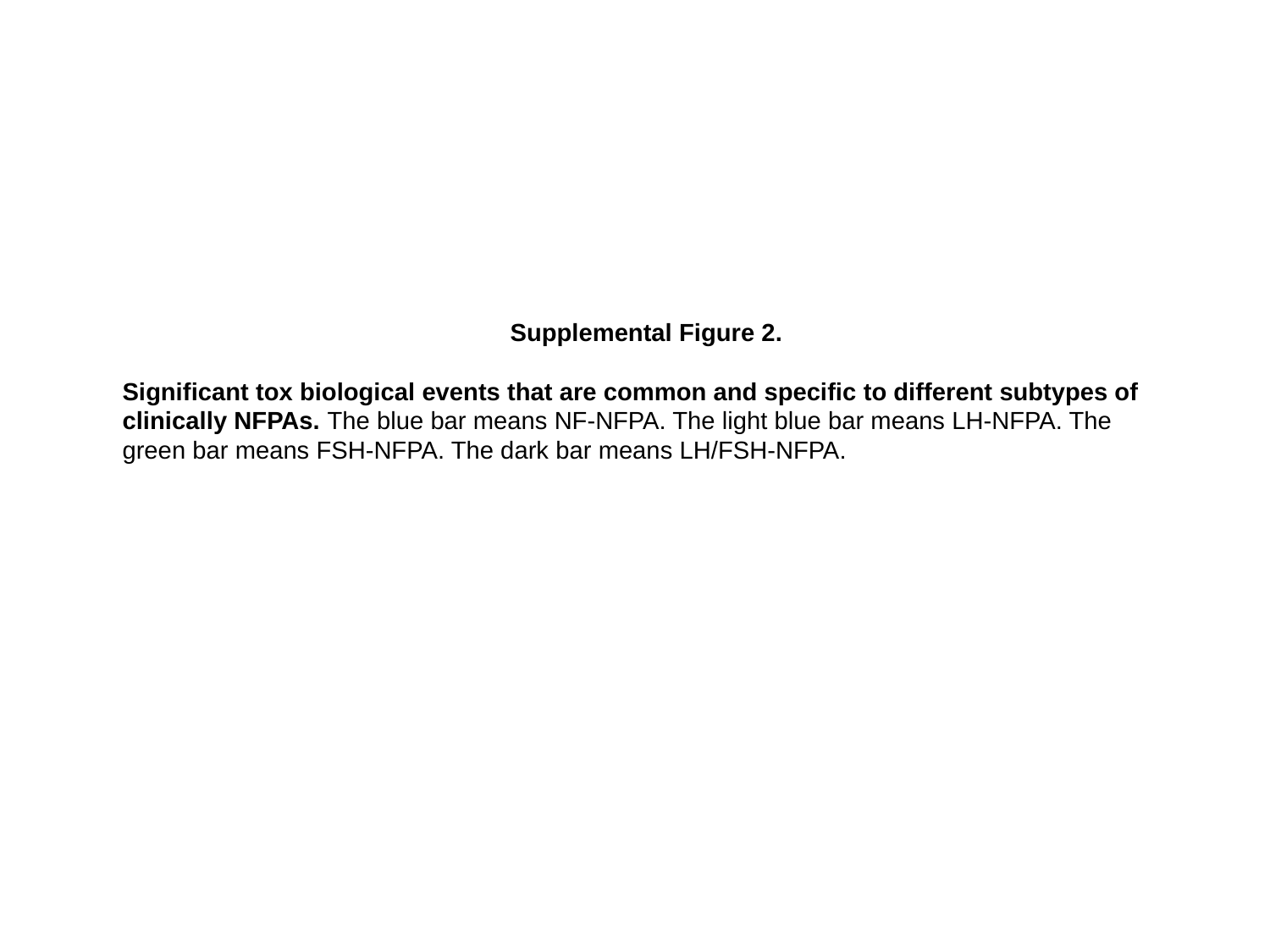

Supplemental Figure 2.
Significant tox biological events that are common and specific to different subtypes of clinically NFPAs. The blue bar means NF-NFPA. The light blue bar means LH-NFPA. The green bar means FSH-NFPA. The dark bar means LH/FSH-NFPA.

## Slide 9
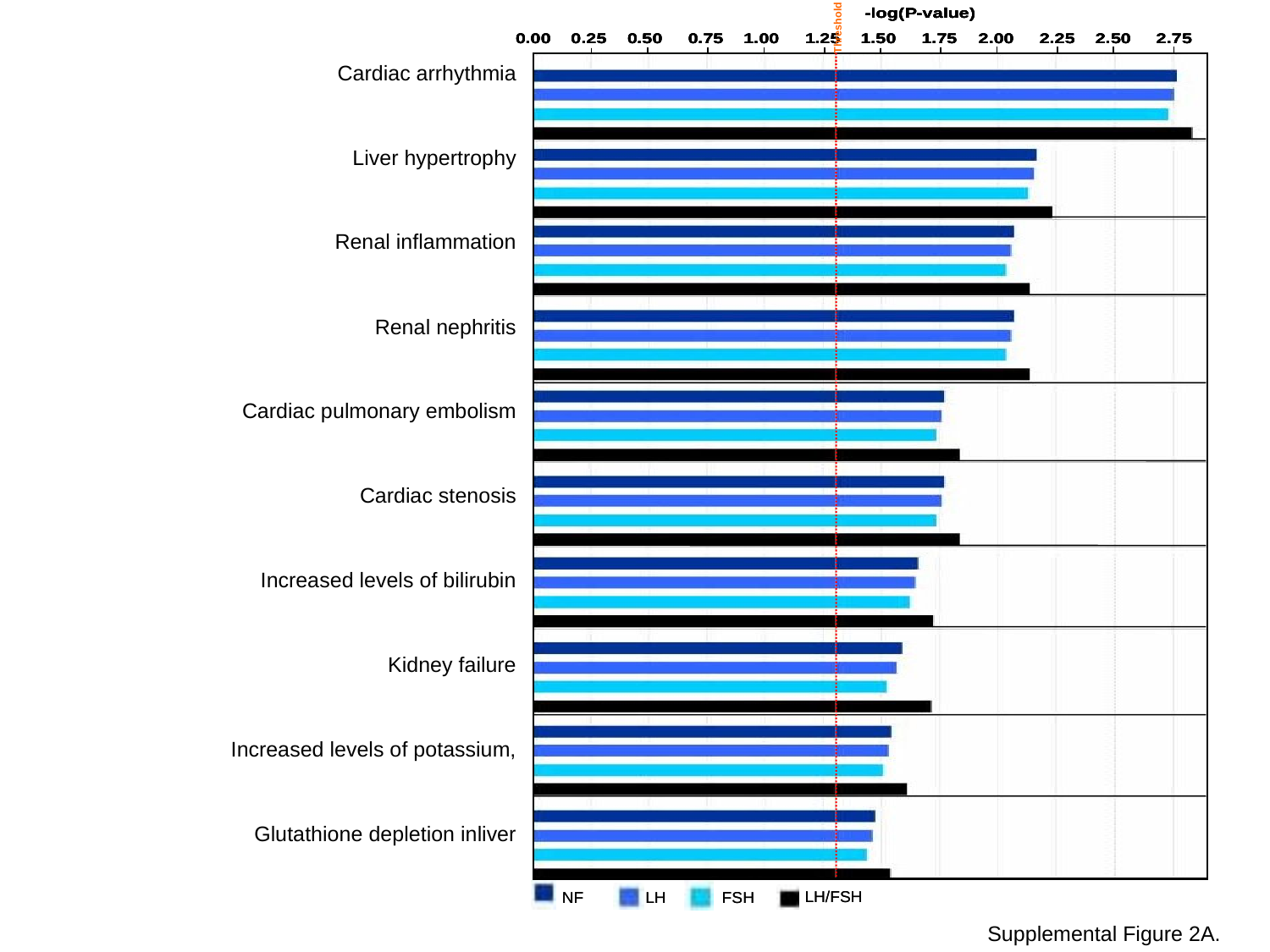

Threshold
Cardiac arrhythmia
Liver hypertrophy
Renal inflammation
Renal nephritis
Cardiac pulmonary embolism
Cardiac stenosis
Increased levels of bilirubin
Kidney failure
Increased levels of potassium,
Glutathione depletion inliver
Supplemental Figure 2A.

## Slide 10
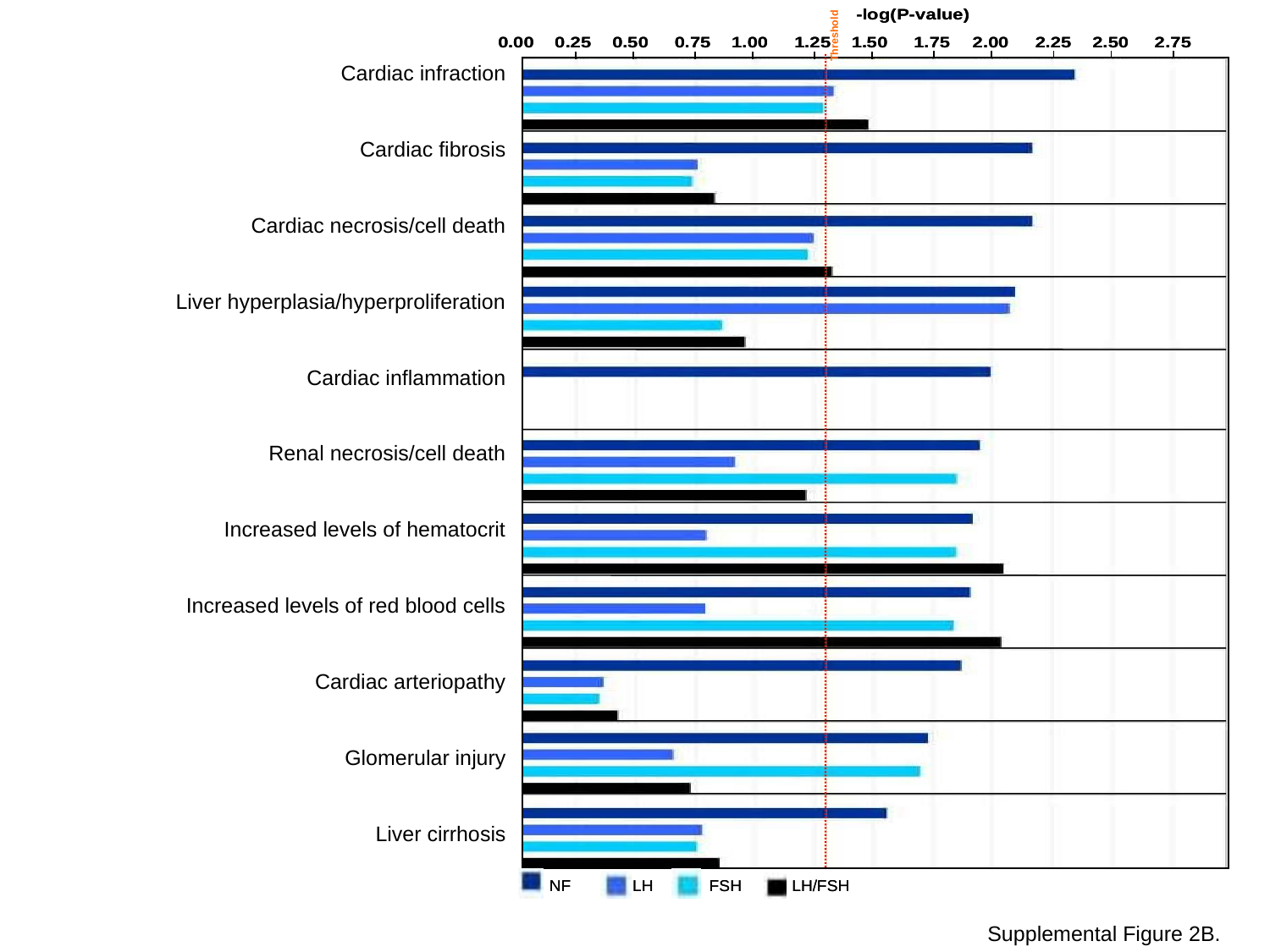

Threshold
Cardiac infraction
Cardiac fibrosis
Cardiac necrosis/cell death
Liver hyperplasia/hyperproliferation
Cardiac inflammation
Renal necrosis/cell death
Increased levels of hematocrit
Increased levels of red blood cells
Cardiac arteriopathy
Glomerular injury
Liver cirrhosis
Supplemental Figure 2B.

## Slide 11
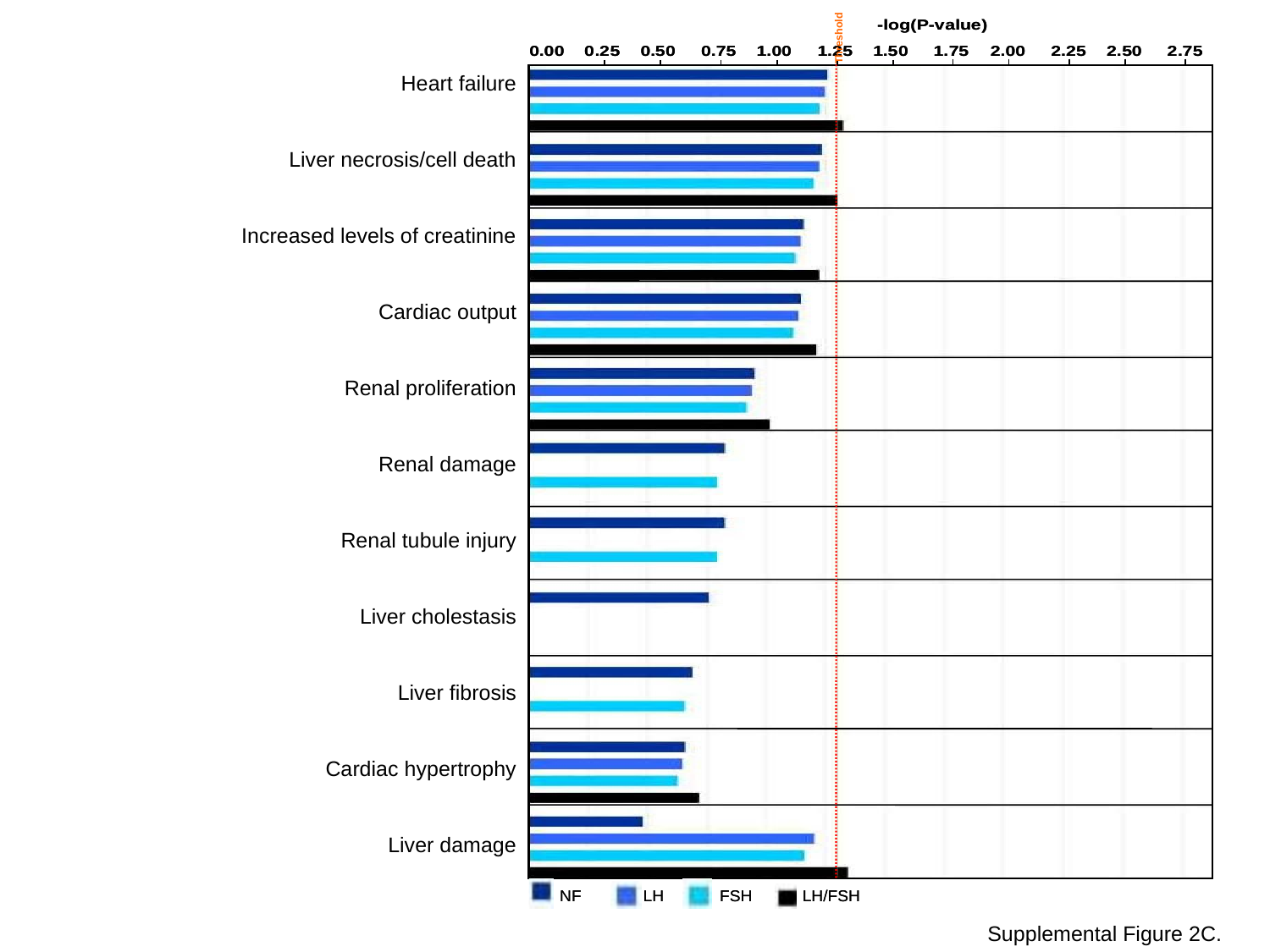

Threshold
Heart failure
Liver necrosis/cell death
Increased levels of creatinine
Cardiac output
Renal proliferation
Renal damage
Renal tubule injury
Liver cholestasis
Liver fibrosis
Cardiac hypertrophy
Liver damage
Supplemental Figure 2C.
